# Endogenous adenosine maintains cartilage homeostasis and exogenous adenosine inhibits osteoarthritis progression

**DOI:** 10.1038/ncomms15019

**Published:** 2017-05-11

**Authors:** Carmen Corciulo, Matin Lendhey, Tuere Wilder, Hanna Schoen, Alexander Samuel Cornelissen, Gregory Chang, Oran D. Kennedy, Bruce N. Cronstein

**Affiliations:** 1 Department of Medicine-Division of Translational Medicine-NYU School of Medicine, 550 First Avenue, New York, New York 10016, USA; 2 Department of Orthopedic Surgery-NYU School of Medicine, 550 First Avenue, New York, New York 10016, USA; 3 Department of Radiology-NYU School of Medicine, 550 First Avenue, New York, New York 10016, USA; 4 Department of Anatomy, the Royal College of Surgeons in Ireland, 123 St Stephens Green, Dublin 2, Ireland; 5 Department of Medicine-Division of Rheumatology-NYU School of Medicine, 550 First Avenue, New York, New York 10016, USA

## Abstract

Osteoarthritis (OA) is characterized by cartilage destruction and chondrocytes have a central role in this process. With age and inflammation chondrocytes have reduced capacity to synthesize and maintain ATP, a molecule important for cartilage homeostasis. Here we show that concentrations of ATP and adenosine, its metabolite, fall after treatment of mouse chondrocytes and rat tibia explants with IL-1β, an inflammatory mediator thought to participate in OA pathogenesis. Mice lacking A2A adenosine receptor (A2AR) or ecto-5′nucleotidase (an enzyme that converts extracellular AMP to adenosine) develop spontaneous OA and chondrocytes lacking A2AR develop an ‘OA phenotype' with increased expression of *Mmp13* and *Col10a1*. Adenosine replacement by intra-articular injection of liposomal suspensions containing adenosine prevents development of OA in rats. These results support the hypothesis that maintaining extracellular adenosine levels is an important homeostatic mechanism, loss of which contributes to the development of OA; targeting adenosine A2A receptors might treat or prevent OA.

## Abstract (web summary)

Osteoarthritis (OA) is a debilitating and destructive joint disease for which disease modifying drugs are not available. Here the authors show that extracellular adenosine signalling via the A2AR receptor on chondrocytes is needed to prevent OA and that liposome-bound adenosine injection can treat the pathology in rats.

Osteoarthritis (OA) is the most common type of arthritis, affecting up to 25% of the population over 65, and 12% of all cases may be caused by prior joint trauma^1^,^2^. Worldwide in its distribution, the incidence of OA increases with age and the resulting pain, loss of joint function and mobility, social isolation, and a broadly reduced quality of life make OA a condition with a high medical and social impact. Current treatment options are less than optimal and do not correct the underlying problem with the result that joint replacement surgery is often the eventual outcome^3^.

OA is characterized by changes in every structure in the joint, including cartilage destruction, synovial inflammation, osteophyte formation, enthesophytes, and significant bony changes^4^. The central player in OA is the chondrocyte, which responds to excess mechanical loading by releasing inflammatory mediators and proteolytic enzymes causing further cartilage damage^5^. In addition, age-related inflammation contributes to the pathogenesis of OA^6^.

Adenosine is an endogenously produced physiological regulator and its intracellular and extracellular concentration is tightly controlled by oxygen consumption, cellular stress and mitochondrial functionality. Extracellular adenosine derives mainly from hydrolysis of ATP (primarily, but not exclusively, by the ectoenzymes CD39 and CD73) and mediates its effects via activation of G-protein-coupled receptors (A1R, A2AR, A2BR and A3R). Adenosine has long been known to regulate inflammation and immune responses^7^,^8^ and work from our lab and others have demonstrated the importance of adenosine and its receptors in osteoblast, osteoclast, and bone marrow homeostasis^9^,^10^,^11^,^12^,^13^,^14^,^15^,^16^,^17^,^18^,^19^,^20^,^21^. Prior studies have suggested that adenosine receptors also regulate chondrocyte physiology and pathology in response to inflammatory stimuli in rodent, equine, bovine and human chondrocytes^22^,^23^,^24^,^25^,^26^ although the specific receptor(s) involved are not identified. Removal of endogenous adenosine (by addition of adenosine deaminase) or blockade of A2AR leads to cartilage degradation in equine cartilage explants^27^, although equine purine metabolism differs from other species as adenosine deaminase, present in lymphocytes, plasma and extracellular fluid of most species, is not present in horse lymphocytes or serum^28^,^29^. A3R stimulation has been reported to diminish OA development in a chemically induced model of OA^30^, principally due to the anti-inflammatory effects of A3R agonists.

Mice lacking A2AR were first developed by Chen *et al*. in 1999 (ref. 31) and, in general, these mice have few obvious defects. Interestingly, they develop osteopenia as a result of an increase in osteoclast number and function and they respond abnormally to a variety of stressors reflecting the loss of the A2AR. However, as these mice age we note that they have increasing difficulty in grabbing food, walking and mating. We asked, therefore, whether the diminished mobility of A2AR-deficient mice might be due to intrinsic joint disease.

We report here that mice lacking A2AR spontaneously develop OA, characterized by altered cartilage composition and loss of cartilage, presence of chondrocyte hypertrophy and osteophytes, and increased subchondral bone density. Rat articular cartilage explants release ATP and adenosine and load bearing in the physiological range induces further increases in extracellular adenosine levels. Interleukin-1β (IL-1β), an inflammatory mediator that contributes to OA, diminishes ATP and adenosine release from cultured chondrocytes. Physiological loading of rat tibia increases adenosine release and is abrogated by IL-1β treatment. Consistent with the role of adenosine in maintaining cartilage homeostasis, mice lacking CD73 also develop signs of OA. Finally, in a rat model of post-traumatic OA, intra-articular injection of liposomal suspensions containing adenosine prevent development of OA and this effect is abrogated by co-injection of an A2AR antagonist, but not an A2BR antagonist. These findings indicate that extracellular adenosine, acting at A2AR, has a critical function in the maintainenance of cartilage homeostasis and that adenosine replacement may be a new approach to treat or prevent OA.

## Results

### Mobility of A2AR KO mice

**Figure 1:**
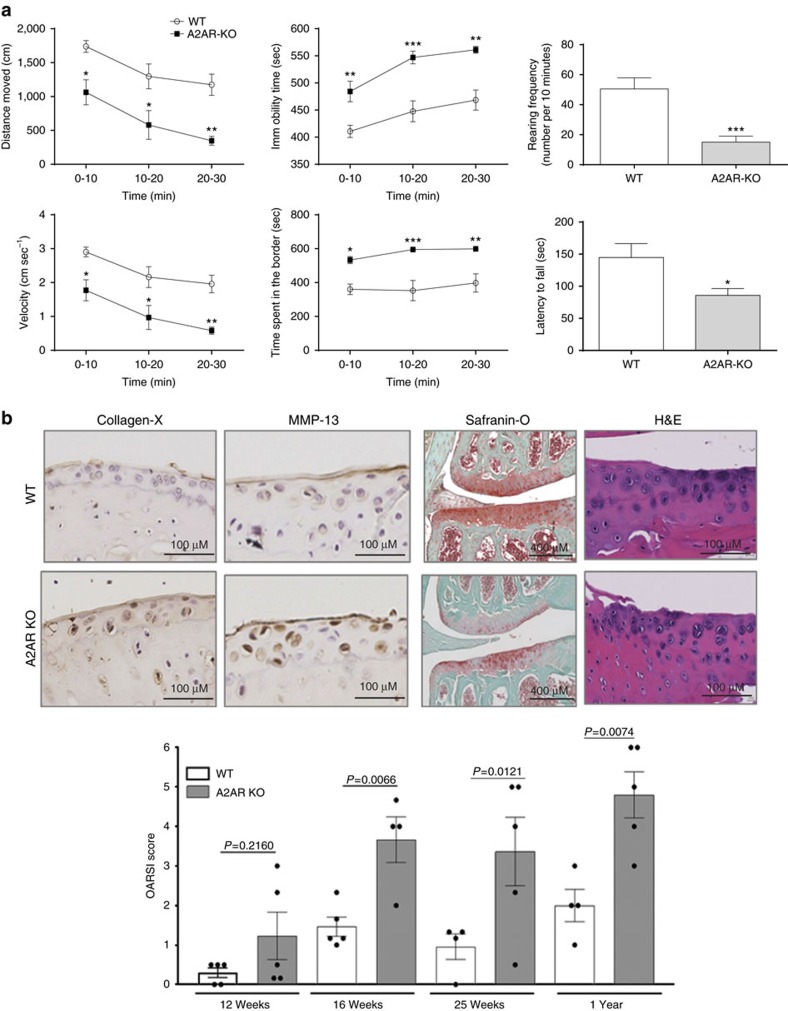
Osteoarthritis in A2AR KO mice. (**a**) Open field and rotarod tests were carried out in 25-week-old WT and A2AR KO mice (*n*=5 for each group) and the digital record analysed, as described in Materials and Methods. Shown are the mean (±s.e.m.) values obtained during each 10 min interval for distance moved, immobility time, rearing frequency, velocity of motion and time spent in the border and the latency to fall in the rotarod test. (**b**) Immunohistologic staining for Collagen X and MMP-13 in a representative section of tibia from a WT and A2AR KO mouse (12 week old mice, original magnification 400 X). Sections from the same mice stained with Safranin O and hematoxylin and eosin (H&E) are shown in the right-hand panels (original magnification 100 X). In the lower panel is plotted the OARSI scores obtained on safranin-O-stained knees, as described in Materials and Methods. Each data point represents the mean (±s.e.m.) of the blinded scores obtained on 4–5 different mice. *P<0.05, **P<0.01, ***P<0.001 versus WT (Student *T*-test or one-way analysis of variance followed by Bonferroni *post hoc* test, as appropriate).

Although the mobility of A2AR knockout (KO) mice appeared to be limited we confirmed that deletion of A2AR leads to loss of mobility by subjecting wild-type (WT) and A2AR KO mice to an open field test to quantitate movement over time. The A2AR KO mice moved a shorter distance than WT mice (Fig. 1a, A2AR KO=664±123 cm versus WT 1,404±129 cm; *P*<0.001 (Student's *T*-test), moved less quickly (velocity for A2AR KO=1.11±0.21 cm s^−1^ versus WT 2.34±0.22 cm s^−1^; *P*<0.001 (Student's *T*-test)) with increased time spent in the border of the cage (A2AR KO=575.5±7.7 s versus WT=370.2±45.6 cm s^−1^; *P*<0.001 (Student's *T*-test)) and immobile (A2AR KO=530.8±11.5 s versus WT 442.3±15.4 s; *P*<0.001 (Student's *T*-test)). The frequency of rearing, a normal mouse exploratory behaviour (measured in the first 10 min of the field test), was also reduced in A2AR KO mice (15±4 times per 10 min, *P*<0.0001 (Student's *T*-test)) compared to WT mice (51±7 times per 10 min). The capacity of the A2AR KO mice to run on the rotarod apparatus with a constant acceleration was also reduced compared to the WT mice (A2AR KO=86±11 s versus WT=145±22 s, *P*<0.01 (Student's *T*-test)).

### μCT analysis of knee joints in A2AR KO and WT mice

Because one potential cause for the difficulty of the A2AR KO mice in mobility, holding food and breeding could be the development of arthritis we examined the knees of A2AR KO mice by μCT. Three dimensional reconstruction of μCT images demonstrated subchondral bony sclerosis and cartilage subsidence in the tibial heads of 8-, 12-, 26- and 52-week-old A2AR KO mice (Supplementary Fig. 1B). Moreover, in the same A2AR KO mice there were osteophytes that increased in size as the animals aged (Supplementary Fig. 1A). No bony or cartilaginous changes were observed in WT mice (Fig. 1b). Moreover μCT analysis revealed a peak of bone volume, trabecular number and thickness in the midshaft bone of WT mice that gradually decreased with age. In contrast, in A2AR KO mice bone does not increase over time and then begins to decline resulting in an osteopenic phenotype once the animals reach adulthood. Similar results were obtained for the bone mineral density values (Supplementary Table 1). In the subchondral area a significant decrease in bone mineral density was observed in the femurs of 8 week old mice and in the tibia and femur of 8 and 12 week old mice (Supplementary Table 2). The cortical area of the subchondral bone showed an initial decrease of the bone volume and density at 8 and 12 weeks and an increase of these parameters in older A2AR KO mice compared to the age-matched WT mice (Supplementary Table 3). The bone and cartilage changes observed were similar in male and female A2AR KO mice.

### Histopathological changes in cartilage of A2AR KO mice

The radiologic appearance of the joints obtained by μCT resembled changes seen in OA; to confirm that the radiologic changes reflected OA in the joints of the A2AR KO mice we examined histologic sections of the knees of these mice. Examination of hematoxylin- and eosin-stained knees revealed progressive reduction in cartilage thickness in A2AR KO mice compared to WT mice; these changes were detectable as early as 12 weeks of age (Fig. 1b). Over time there was increased fibrillation and thinning of cartilage as well, with increased subchondral bone and osteophytes. The chondrocytes present in the cartilage of the A2AR KO mice were increasingly disordered over time, as well. There was significantly less glycosaminoglycan staining in the cartilage of A2AR KO mice by safranin O staining (Fig. 1b) and PAS and trichrome stains further demonstrated loss of sulfated proteoglycans and collagen in the cartilage matrix (Supplementary Fig. 2). These changes were detectable as early as 12 weeks of age. Immunohistochemical staining showed increased MMP-13-positive and collagen X (Fig. 1b), osteopontin- and fibronectin-positive cells in cartilage matrix of the A2AR KO mice starting as early as 12 weeks of age (Supplementary Fig. 2). Finally, a composite score for osteoarthritic changes (OARSI score) showed marked differences between A2AR KO and WT mice, and the differences increased over time. Increased OARSI scores were first detectable at 12 weeks of age. Both male and female A2AR KO mice were affected by OA although the changes were milder in females than males (e.g., OARSI score at 1 year 4.8±0.6 versus 3.2±0.2, males versus females, respectively, *P*<0.05, *n*=4-5 for each (Student's *T*-test)).

### Deletion of A2AR increases MMP-13 and Col10a1 expression

**Figure 2:**
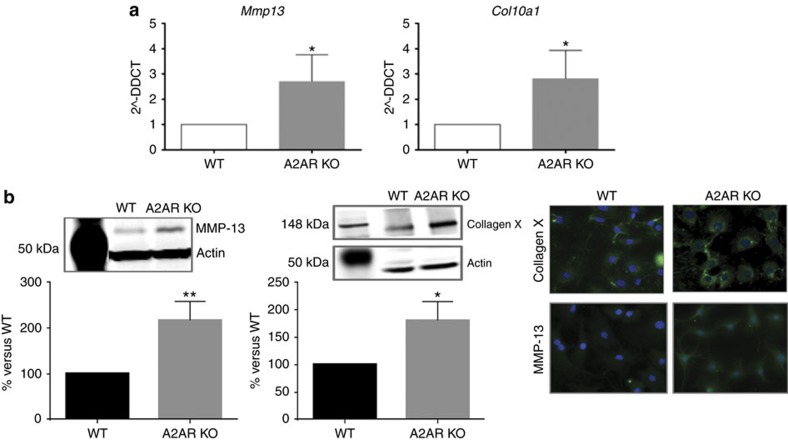
Expression of osteoarthritis markers in primary A2AR-KO and WT chondrocytes. (**a**) Primary chondrocytes were isolated from neonatal WT and A2AR-KO mice. RNA was isolated, reverse transcribed and analysed by real time PCR. Shown are expression levels normalized to GAPDH for *Mmp13* and *Col10a1* levels when compared to levels in WT cells. (**b**) Representative western blots are shown for MMP-13 and collagen X, and quantification of protein bands in the bar graphs below (*n*=4). (**c**) Staining of cell layers shows increases in MMP-13 and collagen X in the A2AR KO chondrocytes compared to the WT. (*n*=4; data are represented as means±s.e.m.). *P<0.05; **P<0.01 versus WT; Student's *T*-test.

In contrast to normal resting chondrocytes, chondrocytes from osteoarthritic cartilage express markers of hypertrophy, for example, *col10a1*, in addition to mediators that participate in the destruction of cartilage, for example, matrix metalloproteases like *mmp13*. As expected, chondrocytes isolated from the cartilage of neonatal WT mice do not express *col10a1* or *mmp13* mRNA or protein (Fig. 2). In contrast, chondrocytes from neonatal A2AR KO mice express both of these markers of OA (Fig. 2). These findings demonstrate that even shortly after birth chondrocytes from A2AR KO mice are already dysregulated and the changes likely contribute to the OA phenotype observed in the A2AR KO mice.

### A2AR expression in human and rat OA

To determine whether loss of A2AR plays a role in human OA we examined A2AR expression on chondrocytes in osteoarthritic cartilage. We observed that A2AR were upregulated in the chondrocytes of patients with OA and appeared to colocalize with expression of MMP-13, a reflection of OA changes in chondrocytes (Supplementary Fig. 3A). Similar results were detected in the PTOA rat model (Supplementary Fig. 3B).

This change was not surprising as, in prior studies, we and others had demonstrated that there is upregulation of both A2AR receptor expression and function following exposure to inflammatory stimuli (IL-1β and tumour necrosis factor (TNF)) which acts as a feedback regulator of inflammation in both murine and human cells^32^,^33^,^34^,^35^,^36^,^37^. One explanation for the difference between A2AR expression in human and murine OA cartilage is that the findings in A2AR KO mice do not reflect OA development in humans. Alternatively, the disparity between human and murine OA cartilage suggests that despite overexpression of A2AR there is diminished ligand for A2AR and resulting loss of A2AR function leading to development of OA.

### Adenosine and ATP release decrease after Il-1β treatment

**Figure 3:**
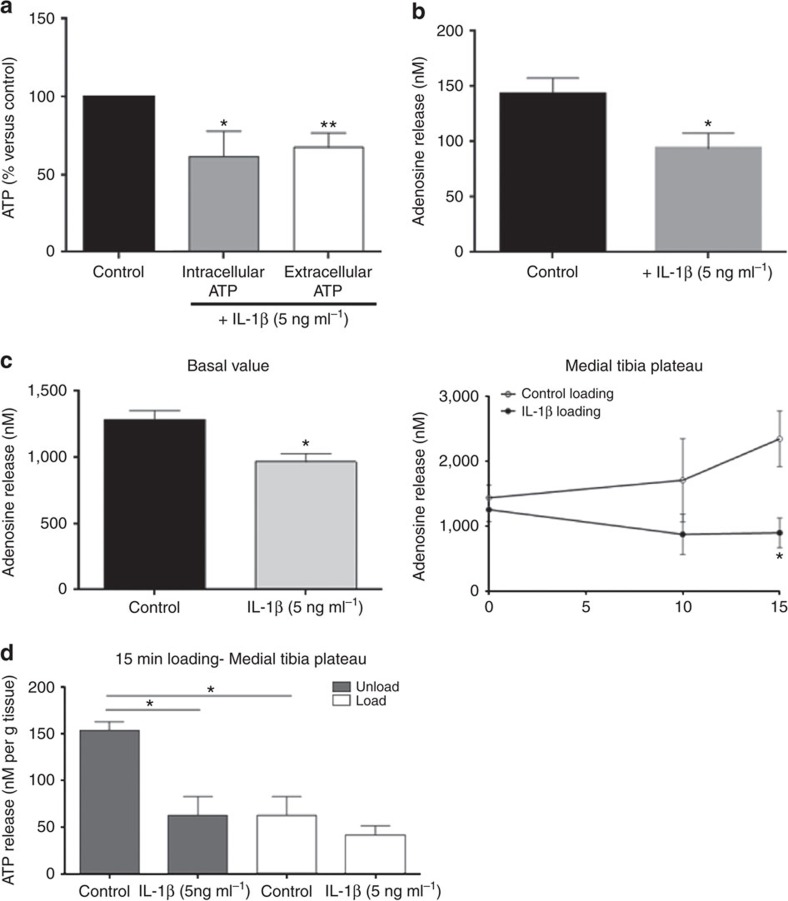
ATP and adenosine decrease after treatment with IL-1β. Treatment of primary murine chondrocytes with IL-1β (5 ng ml^−1^ for 24 h) decreases: (**a**) The intracellular ATP content and the concentration of ATP in the supernatant medium. (**b**) The concentration of adenosine in the supernatant medium. Data are expressed as mean±s.e.m. of 9 experiments performed in duplicate for the adenosine determination and 3 experiments perfomed in duplicate for the ATP assay. Student's *T*-test was performed; *P<0.05, **P<0.01). (**c**) Adenosine levels in supernatants of control and IL-1β-treated rat tibial plateau explants (left) and following mechanical loading of the tibial plateau explants (right, data were analysed for statistical significance by two-way analysis of variance followed by Bonferroni *post hoc* test *P<0.05). The data are expressed as the mean±s.e.m. of five experiments for adenosine release and the adenosine concentration was normalized to the weight of the tibial explant. (**d**) ATP concentration in supernates of tibial explants was measured as described. Each point represents the mean±s.e.m. of three separate determinations.

To test the hypothesis that OA chondrocytes release less adenosine and its precursor, ATP, we quantitated adenosine and ATP release from cultured neonatal mouse chondrocytes and determined whether IL-1β treatment altered this release. We observed that primary mouse chondrocytes release ATP into the extracellular space and that adenosine is present in supernates of cultured chondrocytes (Fig. 3). Treatment of primary mouse chondrocytes with IL-1β (5 ng ml^−1^) for 24 h reduced the level of intracellular ATP (to 61±9% of control, *P*<0.05 (Student's *T*-test); Fig 3a) and the ATP released into the supernates of cultured chondrocytes (to 66±3% of control, *P*<0.001 (Student's *T*-test); Fig 3a). Similarly, IL-1β treatment reduced the adenosine concentration in the supernates of cultured chondrocytes to 65% of control (Control=143±14 nM versus IL-1β=94±14 nM, *P*<0.02, *n*=9 (Student's *T*-test); Fig. 3b).

To understand how IL-1β treatment reduces ATP and adenosine release we examined the effect of IL-1β on expression of ATP transporters (ANKH, pannexin1), the ecto-enzymes involved in conversion of ATP to adenosine (ectonucleotide triphosphate dephorphorylase, ENTPD1 and ecto-5′nucleotidase, NT5E) and the transporters responsible for adenosine transport to and from the extracellular space (nucleoside transporters 1 and 2, SLC29A1 and SLC29A2, respectively). In addition to reducing intracellular levels of ATP, treatment of murine chondrocytes with IL-1β (5 ng ml^−1^ for 24 h) significantly reduced expression of message for all of these molecules (*n*=4 for each experiment, Supplementary Fig. 4).

Because chondrocytes cultured as a monolayer may not behave like chondrocytes embedded within a matrix we determined whether there was a similar reduction in ATP and adenosine release from rat chondrocyte explants. Both ATP and adenosine (Fig. 3a,b) are released from rat tibial plateau explants and treatment of these cartilage explants with IL-1β reduced adenosine release from cells. We next determined the effect of weight loading, within the physiologic range, on ATP and adenosine release from rat cartilage explants and found that there was a marked increase in adenosine release that was abrogated by pre-treatment with IL-1β (1,657±177 nM versus 960±57 nM, respectively, *n*=5; Fig. 3c). Interestingly, after loading of explants there was no detectable change in ATP levels in supernates IL-1β-treated explants from control although there was nearly a 2-fold increase in the adenosine concentration (Fig. 3d). Most likely the increase in adenosine concentration reflects rapid conversion of ATP to adenosine as the cartilage is loaded.

### Reduced capacity to generate extracellular adenosine leads to OA

**Figure 4:**
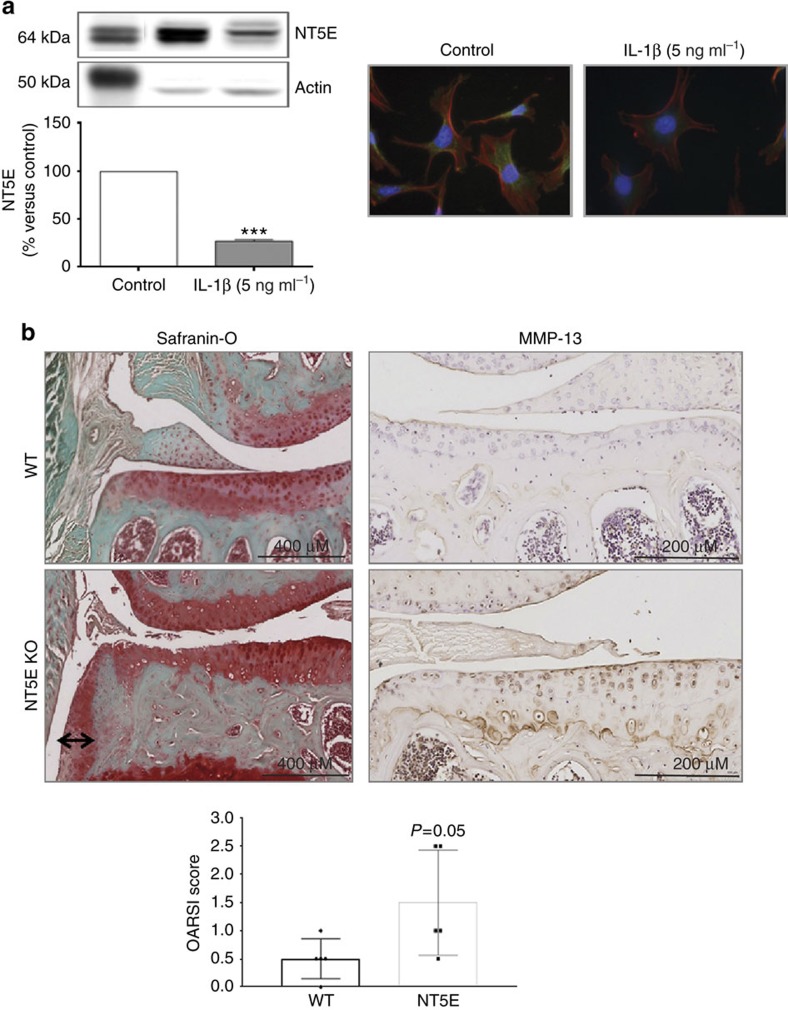
NT5E decreases after IL-1β treatment and NT5E KO mice manifest mild OA. (**a**) 24 h treatment with IL-1β decreases NT5E expression in the total protein content and NT5E membrane expression on the cell membrane. Representative NT5E western blot and protein quantification is shown on the graph on the left side of the figure and on the right are shown representative immunofluorescence photomicrographs of NT5E (green fluorescence) and phalloidin staining (red fluorescence). (**b**) Shown are representative images of knees from 1-year-old NT5E KO mice with cartilage fraying, minimal loss of proteoglycan and increased MMP-13 expression by immunohistochemistry. The black arrow in the safranin-O-stained section indicates an osteophyte.

Multiple transporters and enzymes are involved in the release of ATP from cells and conversion of extracellular adenine nucleotides to adenosine. ANKH and pannexin1 all transport ATP out of the cell^38^,^39^, and while multiple phosphatases can convert ATP to adenine nucleotides and adenosine it is thought that ectoenzymes on the cell surface (ENTPD1 and NT5E) play a critical role in the generation of adenosine available for ligation of adenosine receptors. Other membrane proteins transport adenosine into and out of the cell (nucleoside transporters 1 and 2, SLC29A1 and SLC29A2, respectively). Treatment of murine chondrocytes with IL-1β (5 ng ml^−1^ for 24 h) significantly reduced expression of message for all of these molecules (*n*=4 for each, Supplementary Fig. 4). Moreover the treatment of mouse chondrocytes with IL-1β (5 ng ml^−1^) decreased NT5E protein expression and its localization on chondrocyte surface (Fig. 4a).

Mice lacking ANKH develop severe arthritis at an early age and the joint destruction bears many of the hallmarks of OA, such as osteophytes^40^. Mice lacking ENTPD1 have no changes in their joints (Not shown) but mice lacking NT5E develop mild osteoarthritic changes in their joints (OARSI score=1.5±0.84 versus WT=0.5±0.15) with mild articular cartilage fibrillation, loss of cartilage proteoglycan and osteophytes (Fig. 4b). Interestingly, humans lacking NT5E develop severe arterial calcification and have been noted to have periarticular calcification and arthritis as well^41^,^42^. These results are consistent with the hypothesis that diminished capacity to generate extracellular adenosine contributes to the pathogenesis of OA.

### Liposomal adenosine protects from OA progression in PTOA rats

**Figure 5:**
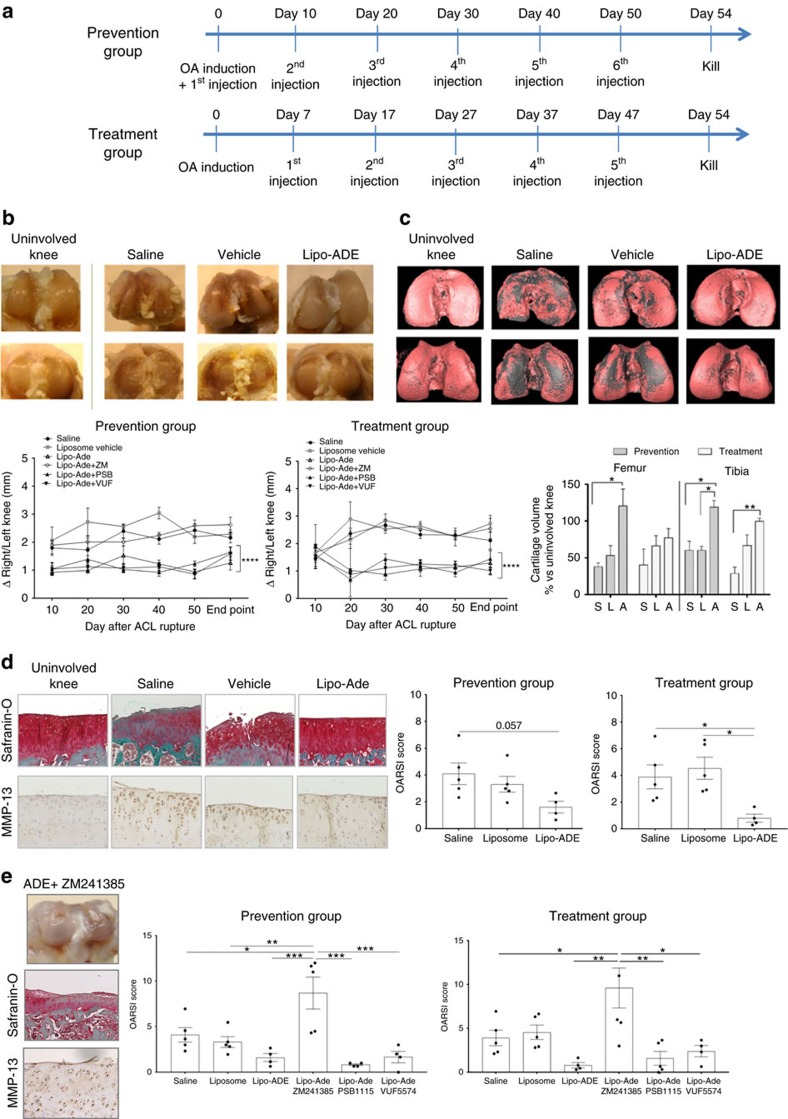
Adenosine prevents and treats OA in a PTOA rat model via A2AR ligation. (**a**) Shown is the experimental design with anterior cruciate ligament disruption on day 0 followed by injection of liposomal adenosine at the indicated times. (**b**) Representative photographs of the gross appearance of the knees of the rats at the time of killing (top row femur, bottom row tibia) and on the bottom is shown knee size measured with a caliper immediately before injection. (**c**) Representative μCT images of hexabrix-imaged cartilage (Top row, femur; bottom row, tibia). The cartilage is pink in this image and underlying bone is grey. In the panel beneath is shown the mean (±s.e.m.) volume of cartilage present in the affected knee expressed as the percentage of the volume of cartilage in the unaffected knee. S, saline-injected; L, empty liposome-injected; A, adenosine-liposome injected. (**d**) Shown are representative safranin-O-stained sections and immunohistologic sections for MMP-13 expression in rat tibial plateaus following ACL rupture. Graphs show the OARSI scores of the knees of the rats studied here. (**e**) Representative gross photograph and photomicrographs of rat knee injected with liposome formulation containing adenosine plus ZM241385 and respective safranin-O staining and MMP-13 immuhohistochemistry. Graphs show the OARSI scores for rat knees treated with adenosine plus adenosine receptor antagonists. Data are expressed as mean±s.e.m. of 5–6 animals for each group and data were analysed for statistical significance by one-way analysis of variance followed by Bonferroni *post hoc* test of differences among various treatments (*P<0.05; **P<0.01, ***P<0.001).

If diminished adenosine levels and A2AR stimulation play a role in the pathogenesis of OA then adenosine repletion might represent one approach to the treatment or prevention of OA. Because adenosine itself has an extremely short half-life (measured in seconds) in blood and other bodily fluids due to the presence of enzymes that degrade adenosine as well as cellular uptake of adenosine^43^,^44^ we formulated adenosine in liposomes to prolong the half-life of adenosine and tested the effect of liposomal adenosine preparations on the development of OA in a rat model of post-traumatic OA. Chondrocytes in rat cartilage express all four adenosine receptors and immunohistochemistry analysis shows an increase of A2AR in OA rats compared to the contralateral healthy knee (Supplementary Fig. 5). Liposomal adenosine, saline or empty liposomes were injected into the affected joint at the time of injury (prevention) or beginning one week later (treatment) and continued in both groups every 10 days for a total of six or five joint injections, respectively (Fig. 5a). Of note, injection of liposomal adenosine almost completely prevented joint swelling in the prevention group and markedly reduced joint swelling in the treatment group over the course of the treatment (Fig. 5b). There was almost complete destruction of the affected tibial cartilage with less destruction in the femoral cartilage in the saline- and liposome-treated rats whereas the liposomal adenosine-treated rats were almost completely protected from cartilage destruction and microCT of hexabrix-stained cartilage confirms these effects (Fig. 5c). In the rats treated with the prevention regimen there was complete preservation of both femoral and tibial cartilage but in the rats treated with the treatment regimen the reduction in cartilage loss did not achieve statistical significance (Fig. 5c). Consistent with the gross and radiologic findings, histologic examination of the joints demonstrated almost complete protection of the joints by liposomal adenosine but not by injections of either saline or empty liposomes (Fig. 5d). In the prevention group treatment with intra-articular injections of saline, empty liposomes and liposomal adenosine resulted in OARSI scores of 4.1±0.8, 3.3±0.6 and 1.6±0.4, respectively (Fig. 5d) and in the treatment group the corresponding OARSI scores were 3.9±0.9, 4.5±0.8 and 0.8±0.3, respectively. Thus, intra-articular administration of liposomal adenosine nearly completely prevented the development of OA.

### The effect of liposomal adenosine is mediated by A2AR

There are multiple adenosine receptors the actions of which can be differentiated by use of appropriate pharmacologic antagonists and agonists. To determine which adenosine receptors are involved in this mechanism, we treated rats with liposomal adenosine plus either an A2AR antagonist (ZM241385), A2BR antagonist (PSB1115) or A3R antagonist (VUF5574.). The co-administration of the A2AR antagonist, but not either the A2BR or A3R antagonist, reversed the effect of liposomal adenosine injections on joint swelling, cartilage degradation or chondrocyte expression of MMP-13. Moreover the OARSI scores of knees from mice treated with liposomal adenosine in the presence of the A2AR antagonist (8.7±1.8 in the prevention group and 9.6±2.3 in the treatment group) were markedly increased compared to those of the knees of rats co-administered either the A2BR (0.8±1.0 for the prevention group and 1.6±0.8 for the treatment group) or A3R antagonist (1.7±0.6 for the prevention group and 2.4±0.6 for the treatment group, Fig. 5d) which did not differ from the values obtained for the rats treated with adenosine-laden liposomes (see above). These results provide strong evidence that A2AR mediates the chondroprotective effects of intraarticular injection of liposomal adenosine.

### NF-κB nuclear translocation

We and others have reported previously that A2AR stimulation inhibits NF-κB translocation in osteoclast precursors^45^,^46^,^47^,^48^,^49^,^50^,^51^,^52^,^53^. Because NF-κB activation, a central event in inflammation, is critical for expression of proteins associated with chondrocyte hypertrophy and cartilage destruction we determined whether A2AR stimulation regulates nuclear translocation of NF-κB in chondrocytes as well. We found that treatment with IL-1β (5 ng ml^−1^) increased NF-κB nuclear translocation, an effect reduced by the A2AR agonist CGS21680 in WT chondrocytes. The A2AR antagonist ZM241385 reversed the effect of CGS21680 on NF-κB translocation. In contrast, CGS21680 did not affect IL-1β-induced nuclear NF-κB translocation in A2AR KO chondrocytes, whether determined by western blotting or by immunofluorescence of whole cells (Supplementary Fig. 6).

## Discussion

The results presented here provide evidence for a critical homeostatic mechanism in cartilage. Chondrocytes release ATP which is converted to adenosine extracellularly; the adenosine that is present prevents the phenotypic changes in chondrocytes associated with development of OA via engagement of A2AR. Disruption of this mechanism, as a result of inflammation, injury or aging with reduction of intracellular and extracellular ATP and extracellular adenosine, leads to phenotypic changes in chondrocytes with greater expression of MMPs or collagens associated with cartilage hypertrophy. Moreover, these studies demonstrate that replacement of adenosine by intra-articular injection of liposomal preparations of adenosine can restore the homeostatic equilibrium to cartilage following injury by engagement of A2AR.

Previous studies of equine cartilage explants demonstrate that removal of extracellular adenosine by treatment with adenosine deaminase significantly increased the release of NO, MMP-13, MMP-3 and GAG, a cartilage matrix breakdown product, and that an A2A agonist reversed this effect. These findings suggested that A2AR are tonically activated in articular chondrocytes preventing a shift in chondrocyte phenotype and chondrocyte-mediated damage to cartilage^27^. Although this finding was very interesting the marked difference between horse and other species with respect to purine metabolism (absent adenosine deaminase in the serum and nearly absent adenosine deaminase in various cell types in horses) suggested that horse tissues might be uniquely dependent on extracellular adenosine to maintain homeostasis^29^. Nonetheless, the results reported here indicate that both mouse and rat chondrocytes and cartilage, respectively, release ATP and adenosine which regulates chondrocyte phenotype in both inflammatory and non-inflammatory settings. Although we did not test the hypothesis that loss of adenosine-mediated regulation of chondrocytes leads to OA-like changes in human chondrocytes or cartilage the similarity of the responses to adenosine of equine cartilage and equine, bovine and human chondrocytes^22^,^23^,^24^,^25^,^26^,^27^ suggests that the changes we observed in rats and mice are generalizable.

Although A2AR KO mice have been observed closely in numerous laboratories for many years their difficulty in mobility and the diminished use of their limbs in eating and mating was not remarked on, nor the cause determined. Here we report that the difficulties in ambulation and other functions are most likely related to the premature development of OA in A2AR KO mice. When examined, the A2AR KO mice have radiologic and histologic characteristics of OA, including the loss of glycosaminoglycans, collagen, and cartilage matrix with fraying, or fibrillation, and destruction of cartilage matrix, increased metalloprotease gene expression and production, chondrocyte hypertrophy, and development of osteophytes. Interestingly, despite the loss of bone volume and substance in A2AR KO mice, previously ascribed to increased osteoclast activity in the bone of these mice^54^, we observed increased subchondral bone density and osteophyte formation, paradoxical findings often observed in patients with OA^55^. Mice heterozygous for loss of A2AR and CD73 were not available for these studies although, because of the effects of inflammation on enhancing A2AR function^56^, it is possible that no difference from WT control mice would have been detected.

In cartilage samples from human OA patients and from PTOA rats, we observed an increase of chondrocyte A2AR expression suggesting that A2AR expression may be a feedback regulatory change in chondrocytes, as previously described for macrophages and endothelium^33^,^36^,^57^. Indeed, expression profiling of chondrocytes from patients with rheumatoid arthritis showed a dramatic increase in A2AR expression as well^58^. Similar to A2AR, the receptor for the parathyroid hormone (PTH), increases in OA patients and in a murine model of OA^59^. The activation of the PTH receptor, a G-protein-coupled receptor that signals via Gs like the A2AR, inhibits articular cartilage degeneration and promotes cartilage and chondrocyte regeneration^59^.

It has recently been appreciated that inflammation plays a central role in the development of OA^60^,^61^ and the findings reported here provide both confirmation of the hypothesis that inflammatory stimuli are central to the development of OA and evidence that tonic suppression of chondrocyte responses to inflammatory stimuli by A2AR ligation are important for joint homeostasis. The demonstration that loss of A2AR leads to premature OA in mice suggests that targeting A2AR might provide a useful approach to the prevention of cartilage deterioration in patients with OA. Indeed, recent studies suggest that methotrexate treatment for patients with symptomatic knee OA provides at least symptomatic relief of OA^62^ and many of the anti-inflammatory effects of methotrexate are mediated by methotrexate-induced increases in extracellular adenosine levels acting at A2AR and A3R^63^. In the therapeutic trials of MTX in OA no attempt was made to evaluate changes in cartilage homeostasis or changes in cartilage destruction and it is unlikely that the short time frame of these studies could have permitted any demonstration of the effects of methotrexate on progressive cartilage loss or other manifestations of OA.

Although we observed a variety of effects of A2AR loss on chondrocytes and cartilage, it is likely that the loss of A2AR in other cells present in the synovium contributed to the development of OA. For example, an increase in osteoclast differentiation and function leads to marked osteopenia in A2AR KO mice^54^. Similarly, adenosine acting at A2AR diminishes expression of a variety of inflammatory cytokines, such as IL-1 and TNF, which likely play a role in the pathogenesis of OA^8^. Thus, tonic A2AR-mediated suppression of inflammatory responses maintains joint homeostasis and one approach to preventing and treating OA is to target A2AR. Nonetheless, testing of conditional A2AR knockouts and selective A2AR knockout on development of OA will yield a more nuanced understanding of the basis of the spontaneous OA in the A2AR knockout mice.

In our studies we used a liposomal formulation to prolong the presence of adenosine in the joint. Indeed, preliminary studies in which we injected an aqueous solution of an A2AR agonist into joints had no effect on joint swelling or development of structural changes in the joint after anterior cruciate ligament disruption (C.C. and B.N.C., unpublished observation). We administered the liposomal adenosine preparations in both treatment and prevention groups of rats and prevented or treated joint swelling similarly. We were surprised to observe that there was less structural damage to the joints, as reflected by a better OARSI score, in the rats in which the initial injection of liposomal adenosine was delayed for a week after knee injury. The likely explanation for this effect is that the initial spike in inflammatory cytokines in the joint following injury of the knee which leads to chondrocyte expression of enzymes capable of destroying cartilage also leads to enhanced expression and function of A2AR in the injured tissue and, thus, a greater adenosine response.

It has long been appreciated that ATP levels are decreased in OA chondrocytes and this reduction in cellular ATP content may result from the effects of age, injury or inflammation on mitochondria^6^. Our results are consistent with these observations and suggest how reduced intracellular ATP in chondrocytes could lead to diminished ATP release into the extracellular space resulting in diminished resistance to the phenotypic changes in chondrocytes that are associated with development of OA. Nonetheless, the reduction in ATP release observed here is greater than the reduction in intracellular ATP levels suggesting the possibility that there is also a reduction in the capacity of chondrocytes to export ATP. There are a number of transporters that can export ATP to the extracellular space including ANKH and pannexins^38^,^39^ and ANKH expression is markedly reduced after exposure to IL-1 (ref. 64) although the effect of injury or inflammatory mediators on pannexin-1 or pannexin-3 expression and function is less well established. Interestingly, loss of pannexin-3 is protective in murine models of OA suggesting that this protein is not involved in the phenomena studied here^65^. The observation that mice lacking expression of ANKH develop arthritis consistent with OA at an early age further supports the hypothesis that extracellular adenosine, derived from ATP, plays a homeostatic role in cartilage.

Further evidence for the importance of adenosine in the maintenance of cartilage and joint homeostasis is provided by the spontaneous OA observed in NT5E KO mice. This ecto-enzyme catalyses the hydrolysis of AMP to adenosine and prior studies have demonstrated that the loss of CD73 activity leads to exaggerated inflammatory responses^66^. Recently patients lacking CD73 have been described and while diffuse large artery calcification dominates the clinical picture nearly all of these patients suffer from a poorly characterized arthritis with associated periarticular calcification^41^,^42^. The rheumatic manifestations observed in these patients are consistent with the notion that relative deficiencies in adenosine are deleterious for the structures of the joint.

Another ectoenzyme, ENTPD1, which catalyses the hydrolysis of ATP and ADP to AMP, also plays an important role in endogenous suppression of inflammation although there are a number of other extracellular phosphatases capable of hydrolysing ATP, such as tissue non-specific alkaline phosphatase^66^. Although we were surprised that ENTPD1 KO mice did not develop OA it is likely that these other phosphatases hydrolysed sufficient ATP to adenosine to maintain homeostasis.

**Figure 6:**
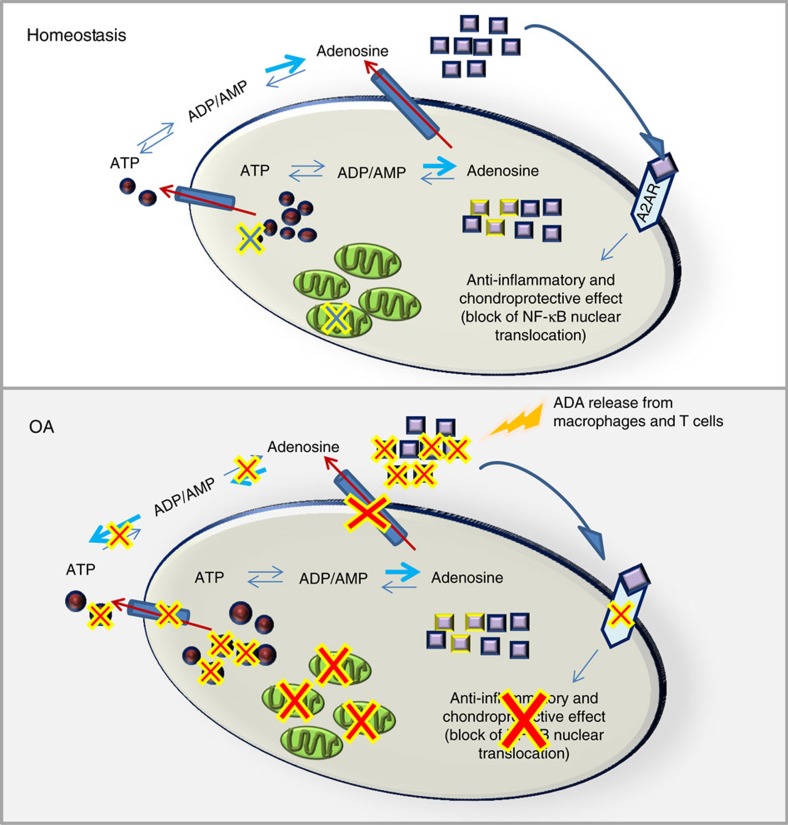
Endogenous adenosine maintains cartilage homeostasis. Here we provide a conceptual model of the role of adenosine production and adenosine receptor ligation in inflammation and in the pathogenesis of OA. Adenosine is generated which ligates A2AR maintaining homeostasis in cartilage after mechanical loading and other stresses. A2AR ligation exerts an anti-inflammatory and chondroprotective effect by blocking NF-κB nuclear translocation and promoting production of growth factors. During OA progression a decrease of adenosine in the extracellular space occurs due partially to IL-1β effect on ATP and adenosine transporters and the enzymes involved in ATP/ADP catabolism. Moreover, decreased mitochondrial number, observed in OA chondrocytes, contributes to diminished cellular ATP levels. Moreover, production and release of adenosine deaminase by inflammatory cells can exacerbate the progression of OA by further reduction of adenosine levels (unpublished figure created by C.C.).

We conclude that adenosine, acting at A2AR, is an important homeostatic regulator of chondrocytes and cartilage and adenosine repletion may represent a novel approach to treating OA (Fig. 6).

## Methods

### Materials

ZM241385 (A2AR antagonist), PSB1115 (A2BR antagonist) and VUF5574 (A3R antagonist) were obtained from TOCRIS (MI, USA). Mouse and rat recombinant IL-1β was obtained from R&D Systems (MN, USA). Antibodies: Rb-MMP-13 (ab39012), Rb-Osteopontin (ab8448), Rb-Fibronectin (ab2413), m-A2A adenosine receptor (ab115250), Rb-A2B adenosine receptor (ab135865), were purchased from ABCAM (MA, USA); Rb-A1 adenosine receptor (AP01303PU-N) was purchased from Acris GmbH (MD, USA); (Rb- NT5E/CD73 (13160) from Cell Signaling (MA, USA); Rb-Collagen-X (234196) from Millipore (MA, USA); Rb-A3 adenosine receptor (sc-13938) and secondary antibody HRP conjugate were obtained from SantaCruz (CA, USA). Hexabrix was purchased from Guerbet (IN, USA). Paraformaldehyde (PFA) 32% was obtained from Electron Microscopy Sciences (PA, USA). RIPA buffer, EDTA, bovine serum albumin, SIGMA*FAST* 3,3′-Diaminobenzidine tablets, Anti-Rabbit IgG–FITC antibody, Anti-Rabbit IgG–TRITC antibody, Amphotericin B, cholesterol, phosphatydil choline from egg yolk, ethanol, glycerol, adenosine and ascorbic acid, murine primer sequences (Tab. 1) were purchased from Sigma-Aldrich (MO, USA). Alexa Fluor 555 phalloidin, DMEM-F12, DMEM, penicillin-streptomycin and fetal bovine serum were purchased from Life technology (NY, USA). ATP determination kit was purchased from Thermo Fisher (MA, USA). The kit for RNA extraction was purchased from Qiagen (CA, USA). The reverse transcription kit was purchased from Applied Biosystem (CA, USA). The Brilliant FAST SYBR Green were obtained from Agilent Technologies (CA, USA). NE-PER kit was purchased from Thermo Scientific.

### Animals

Mice and rats employed in this study were kept under regular lighting conditions (12 h light/dark cycles) and given food and water ad libitum. Adenosine A2A receptor knockout (A2AR KO) mice and CD73 knockout (CD73 KO) mice, both bred on a C57BL/6 backgound, were kindly provided respectively by Dr Jiang Fan Chen (Boston University School of Medicine, Boston, MA) and Dr Linda Thompson (Oklahoma Medical Research Foundation, Oklahoma City, OK). Male and female A2AR KO and CD73 KO mice were bred onto a C57BL/6 background (≥10 backcrosses). Mice were bred and raised in the same facility (Taconic Bioscence), receiving the same food and care. At the time of the experiments mice were transferred to the NYUSoM Medical Center animal facility. Genotyping was performed by PCR, as previously reported^67^. WT and A2AR KO newborn mice, both males and females, were used for chondrocyte extraction from knee joint. Mice were killed by CO_2_ narcosis.

Sprague Dawly male rats were used in the experimental OA induction (*n*=5–6 for each group) and for tibia collection in the *ex vivo* tibia loading experiment (*n*=10).

All protocols for experimental procedures involving the use of animals were approved by the New York University School of Medicine Institutional Animal Care and Use Committee.

### Motor test

To test for and quantitate any motor impairment, two different motor tests were performed on WT and A2AR KO 25 weeks old mice. Mice (*n*=5 for each group) were acclimated in the apparatus for 1 h before the test.

Spontaneous locomotor activity was analysed using the open-field test. Mice were placed into the center of a chamber 25 × 25 × 25 cm to allow free exploration. The experiments were performed for 30 min on 2 consecutive days. Total travel distance, velocity, time spent in the border and immobility time were measured by computerized analysis at 10 min intervals. The number of rearings was quantitated in the first 10 min interval. Mice behaviour was recorded and videos were analysed by use of EthoVision XT software (Noldus, The Netherlands).

Forced locomotor activity was tested by using the rotarod test. Mice were placed on a rotarod apparatus (AccuRotor Rota Rod -Accuscan Instruments, Columbus, OH) and tested for 5 min with a constant acceleration from 4 to 40 r.p.m. The latency to fall was registered for each animal. To exclude differences in learning skills between the two groups of mice, each group was assessed over three trials per day for 2 consecutive days. Mice were given a 30 min inter-trial rest interval.

### Tibial explant loading experiments

14 to 19 week old male Sprague-Dawly rats were killed and their tibias were collected. The soft tissue was removed from the bone and the proximal tibia was cut in the growth plate region by using a Low Speed Diamond Wheel Saw- Model 650 (South Bay Technologies, CA, USA). Tibia surface was cut separating the medial and the lateral condyle. Samples were incubated in media containing DMEM-F12, FBS 10%, 100 μg ml^−1^ of ascorbic acid, 2 mM of L-glutamate, 1% of Penicillin, Streptomycin and amphotericin. The explants were incubated with or without IL-1β (5 ng ml^−1^) for 24 h. After incubation the samples were placed in a glass plate in new media without FBS. The loading was programmed using the block waveform feature in Wintest 7 software of BOSE Electroforce 3200 system (Bose, Inc., MN, USA). The specimens were preloaded with uniaxial compressive load that was applied at 0.005 mm s^−1^ loading rate under displacement control up to 0.01 mm. Samples undergo cyclic load at 2 Hz for 1,800 cycles at 1.5N compressive load under load control. Dwell for 2,700 s immediately after the end of 1,800th cycle. Media samples were collected after 1, 10 and 15 min during loading and 15 and 30 min after loading. Samples collected for ATP assay were stored immediately on ice. Samples used for adenosine extraction were collected in tubes containing Trichloroacetic acid (1:1, v/v).

### PTOA induction in rats and liposomal adenosine treatment

The post-traumatic OA (PTOA) model used is a non-invasive method for inducing anterior cruciate ligament (ACL) rupture in rat knees *in vivo* with a single load of tibial compression. We chose this model in rats because of the technical ease in injecting the knees of these animals and because correction of an induced lesion would better mimic the situation in humans. The procedure was performed under anesthesia (1–3% isoflurane) as previously described^68^. All experimental groups of rats, as described below, consisted of 3 rats and each experimental group was repeated once (total of 6 rats per experimental group). This number of rats was chosen because larger group sizes led to operator overload and diminished quality of results. To have a >90% power to detect a 70% reduction in OARSI score using an analysis of variance with repeated measures, with an α error probability of 0.05 and three groups, we will need a minimum of six animals for each condition. Animals were not randomized for these studies.

Rats were treated with intra-articular injections of 100 μl of a liposomal suspension containing a high concentration of adenosine (10 mg kg^−1^), empty liposomes or with saline for 8 weeks. In some experiments rats were injected intraarticularly with 100 μl of liposomal suspensions of adenosine plus ZM241385 (1 mg kg^−1^), adenosine plus PSB1115 (1 mg kg^−1^), adenosine plus VUF5574 (1 mg kg^−1^). The animals were separated into two main cohorts, the prevention group received injections commencing immediately after ACL rupture, and the treatment group received the first injection 7 days after ACL rupture. Injections were performed every 10 days thereafter in both groups. Knee swelling and weight in the rats were measured before every injection. At the end of the experiment rats were killed and both legs were collected for immunohistochemistry and micro-computed tomography (μCT) analysis.

### Liposome preparation

Liposomes were prepared fresh the day before injection. Ethanol was added to soybean oil containing adenosine, or adenosine plus adenosine receptor antagonists. The lipid phase containing phosphatidyl choline and cholesterol (1:0.5 by molar ratio) was added to the previous solution and emulsified at 15,000 r.p.m. for 10 min. Saline along with glycerin was then added to the lipid phase and was homogenized at 15,000 r.p.m. for 20 min followed by sonication for 1 min at 100% duty cycle.

### Measurement of adenosine concentration by HPLC analysis

To measure adenosine content in the culture medium, 400 μl of the samples were added to a similar volume of thrichloroacetic acid (10% v/v) and then extracted with Freon-octylamine. The HPLC separation of adenosine was achieved by applying the samples to a C18μ Bondapack column (Water Chromatohaphy, Div., Milford, MA) and eluted with a 40% linear gradient of 0.01 M ammonium phosphate (pH 5.5) and methanol with a 1.5 ml min^−1^ flow rate. Adenosine was identified by retention time and the characteristic spectrum of ultraviolet absorbance. The adenosine concentration was determined by comparison to standards.

To test adenosine retention in liposomes, liposome particles were centrifuged at 100,000 g in an ultracentrifuge (Optima L-90K Beckman Coulter) for 15 min at 4 °C, free adenosine was collected from the supernate and different dilutions were tested by HPLC. 73% of the adenosine was retained in the liposomes.

### Histology and immunohistochemistry

After killing, both hind legs were excised from WT and A2AR KO mice (4 animals for each group). Left hind limbs, assigned to immunohistochemistry analysis, were cleaned of soft tissue, placed into 4% PFA for 48 h, and decalcified in 10% EDTA for 4 weeks. Paraffin-embedded histological sections (5 μm) were cut, mounted and prepared for analysis with hematoxylin and eosin and Safranin O/Fast green staining to assay different cartilage components. Collagen X, MMP-13 were detected in cartilage by immunohistochemistry. Briefly, joint sections were deparaffinized by xylene and re-hydrated in decreasing ethanol concentrations. Sections were depleted of endogenous peroxidase activity with 3% H_2_O_2_ in methanol, then blocked with PBST containing bovine serum albumin (1%) and FBS (5%) for 60 min. Sections were incubated overnight with rabbit antibody (1:200 dilution) specific for each protein under study. After rinsing with phosphate-buffered saline (PBS), horseradish peroxidase (HRP)-conjugated secondary antibody was applied and stained with diaminobenzidine (DAB) kit. Slides were scanned using a Leica microscope equipped with Slidepath Digital Image Hub version 3.0 Software. Assessment of OA was performed by evaluation of Safranin-O stained slides in a blinded fashion. OARSI score was determined blindly as previously described^69^. Briefly, for histologic scoring, slides were stained using Safranin-O Fast Green technique. The OA severity is determined by using a 0–6 scoring system: 0 for normal cartilage; 0.5 in case of loss of Safranin-O without structural changes; 1 for small fibrillation without loss of cartilage; 2 when vertical clefts were present; 3, 4, 5 and 6 when vertical clefts and erosion covered <25%, 25–50%, 50–75%, >75% of the articular surface, respectively.

For rat experiments, after killing both legs were excised and fixed with 4% PFA for 48 h and then preserved in 70% ethanol. After μCT analysis the samples were washed with PBS and decalcified in 10% EDTA for 4 weeks. Histology and immunohistochemistry were performed as previously described and OARSI score determined blindly for each specimen taking into account the severity of cartilage degradation, cartilage calcification, presence of osteophytes and their size^70^.

Paraffin sections of human cartilage from a bone excised were supplied by the BioRepository Center of the NYU Langone Medical Center and by National Disease Research Interchange (Philadelphia, USA). Samples were collected from deidentified specimens collected at the time of joint replacement or specimens of normal cartilage were purchased from the National Disease Research Interchange.

The cartilage was collected from two OA patients undergoing total knee replacement surgery and provided without identifiers other than gender and age. Following incubation with primary antibodies slides were incubated with anti-rabbit FITC and anti-mouse TRITC, and imaged under fluorescence microscopy.

### Mouse bone evaluation by μCT

After killing right hind limbs from mice were disarticulated and used for morphology and measurement of bone features by μCT. After fixation in 70% ethanol, femora and tibiae of each sample were scanned with a SKYSCAN-1172 instrument and analysed for subchondral bone density. Briefly, serial 12.5-μm tomographic images were acquired at the condition of 70 kV and 113 mA. Constant thresholds (200) were performed in binary images to segment bone from bone marrow. 2D and 3D images were obtained and the region of interest (ROI) in subchondral bone was defined as a sclerotic area contouring from the bone surface above the growth plate.

### μCT cartilage examination

After washing with PBS, rat knees (femoral and tibial surfaces, *n*=3 for each group) were incubated in PBS containing the ionic contrast agent Hexabrix^r^ (40% v/v) for 6 h. All joints were evaluated in a (16 mm) scanning tube providing a volex size of 10.5 μm and scanned at 55 kV, 181 μA, 110 min of acquisition time. During scanning the samples were wrapped in paper soaked in PBS to avoid dehydration.

### Mouse primary chondrocyte extraction and culture

Articular chondrocytes were obtained from C57BL/6 WT or A2AKO newborn mice following a protocol previously described^71^. Primary chondrocytes (80% confluence) were starved for 14 h. Cells were treated for 24 h with mouse recombinant IL-1β (5 ng ml^−1^) in DMEM containing 10%FBS and 1% Penicillin-Steptomycin. For intracellular ATP assay, cells were collected and lysed with RIPA buffer containing proteases and phosphatase inhibitors. For extracellular ATP and adenosine assays, complete media was replaced with DMEM without FBS and samples were collected after 10 min. ATP was assayed using a bioluminescent ATP determination kit following the manufacturer's instructions. Adenosine was extracted and tested by HPLC as previously described. ATP and adenosine data were normalized following protein quantification.

### Protein extraction and western blotting assay

After cell treatments, the total protein extracts were collected and stored at −80 °C. Total protein fractions were quantified using the BCA kit (Thermo Scientific). For the evaluation of NF-κB nuclear translocation, cells were collected after 10 min of treatment. Then cells were collected and nuclei and cytosolic protein componetns were separated using NE-PER kit (Thermo Scientific) following the manufacter's protocol.

Western blotting was performed by electrophoresing 10 μg ml^−1^ protein through a 10% polyacrylamide gel followed by transfer of proteins to nitrocellulose membranes. Nitrocellulose membranes were incubated overnight at 4 °C with the specific primary antibody (1:1,000), and after washing, incubated with goat anti-rabbit IRDye 800 CW and goat anti-mouse IRDye 680 RD (1:5,000). Membranes were scanned with Li-cor Odyssey equipment and the intensities of the protein bands were quantified by densitometric analysis using Image Studio 2.0.38 software.

### Reverse transcription and Real Time PCR

RNA extraction was performed from mouse primary chondrocytes using RNeasy Mini Kit (Qiagen, Invitrogen) and QIAshredder colums (Qiagen, Invitrogen), following the manufacturer's protocol. RNA reverse transcription was performed using the MuLV Reverse Transcriptase PCR Kit (Applied Biosystems). After RNA reverse transcription to cDNA, real time PCR reactions were performed for a relative quantification of COL10a1 (forward: 5′-TTCTGCTGCTAATGTTCTTGACC-3′; reverse: 5′-GGGATGAAGTATTGTGTCTTGGG-3′), Mmp-13 (forward: 5′-TGTTTGCAGAGCACTACTTGAA-3′; reverse: 5′-CAGTCACCTCTAAGCCAAAGAAA-3′), ENTPD1 (forward:5′-ACAAGGGCTGCGAGATAAGA-3′; reverse: 5′-CCACCCAGACCTGTTGACTT-3′), NT5E (forward: CAAATCCCACACAACCACTG-3′; reverse: 5′-TGCTCACTTGGTCACAGGAC-3′), PANX1 (forward: 5′-CCACCGAGCCCAAGTTCAA-3′; reverse: 5′-CCGGGTTGTTGAGTGTTACAG-3′), SLC29A1 (forward: 5′-CCAGTGGTTCTGAGCTGTCA-3′; reverse: 5′-CTGTTGGTGGGTGGAGAGTT-3′), SLC29A2 (forward: 5′-GCTGGGTACCATGCCTTCTA-3′; reverse: 5′-CCACACAGGGTGTGATGAAG-3′) and ANKH (forward: 5′-CAAGAGAGACAGGGCCAAAG-3′; reverse: 5′-AAGGCAGCGAGATACAGGAA-3′) performed on a Stratagene Mx3005P (Agilent Technologies) with Brilliant SYBR Green Kit QPCR Master Mix (Stratagene, Agilent Technologies), according to the manufacturer's protocol.

### Immunofluorescence

Cells were plated in 8-well chamber slides and, after the appropriate treatments, were washed with cold PBS and fixed with cold methanol (10 min). Cells were permeabilized using a solution of PBS containing Triton 0.25% for 10 min. After 3 washes for 5 min each, a blocking solution (FBS 5%, BSA 1% in PBST) was added to the cells for 1 h. Cells were incubated with primary antibody against collagen-X, MMP-13, CD73 or NF-κB antibody overnight. Cells were washed 3 times for 5 min each with PBS and incubated with the secondary antibody FITC conjugate (1:200 in PBST) for 1 h and with with 0.5 μg ml^−1^ of TRIC-labelled Phalloidin for 30 min. After 3 washes of 5 min each, a cover slide was applied to the slide witha mounting media containing DAPI. Immunfluorescence was revealed by the Nikon Eclipse Ni fluorescence compound microscope.

### Data analysis

μCT analysis of bone and cartilage volume was performed using CTvox software to reconstruct 2D and 3D images and to calculate various bone characteristics. Amira software (FEI, Oregon-USA) was used to reconstruct mice and rat joints from μCT data based on differential density of bone and Hexabrix-treated cartilage. Statistical significance for differences between groups was determined using Student's *T*-test, two-way or one-way analysis of variance, as appropriate, using GraphPad software (GraphPad, San Diego, CA). If the overall differences were significant (F<0.05) then differences between groups were analysed by Bonferroni *post hoc* testing.

### Data availability

The data that support the findings of this study are available from the corresponding author on request.

## Additional information

**How to cite this article:** Corciulo, C. *et al*. Endogenous adenosine maintains cartilage homeostasis and exogenous adenosine inhibits osteoarthritis progression. *Nat. Commun.* **8**, 15019 doi: 10.1038/ncomms15019 (2017).

**Publisher's note:** Springer Nature remains neutral with regard to jurisdictional claims in published maps and institutional affiliations.

## Supplementary Material

Supplementary Information Supplementary Figures and Supplementary Tables
