# Supplementary material for: Endogenous adenosine maintains cartilage homeostasis and exogenous adenosine inhibits osteoarthritis progression
Source: Nat Commun. 2017 May 11;8:15019. doi: 10.1038/ncomms15019 (PMC5437286; doi:10.1038/ncomms15019)
Supplement: Supplementary Information — Supplementary Figures and Supplementary Tables [file ncomms15019-s1.pdf]

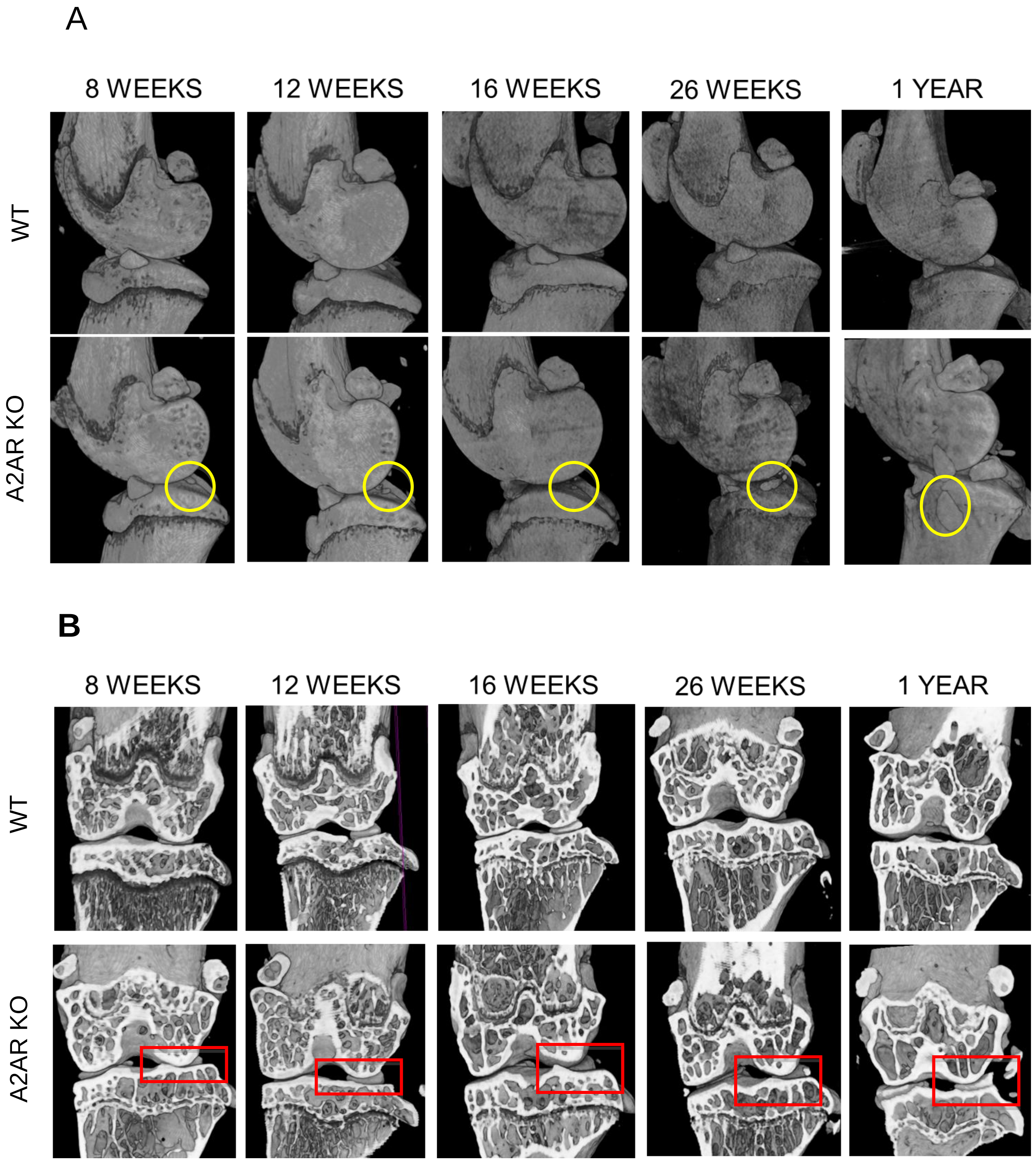
A
8 WEEKS
12 WEEKS
16 WEEKS
26 WEEKS
1 YEAR
WT
A2AR KO
B
8 WEEKS
12 WEEKS
16 WEEKS
26 WEEKS
1 YEAR
WT
A2AR KO

Supplementary Figure 1 - Representative 3D reconstruction of μCT data and their sagittal section of WT and A2ARKO mice at different ages. 3D reconstruction shows presence of osteophytes in femur and tibia of A2ARKO mice (A, yellow circle). In the sagittal section it can be noticed the initial decrease of the cortical subchondral bone in young A2ARKO mice (8-12 weeks), compare to the aged matched WT mice, and the thickening in the adult age (B, red rectangle).

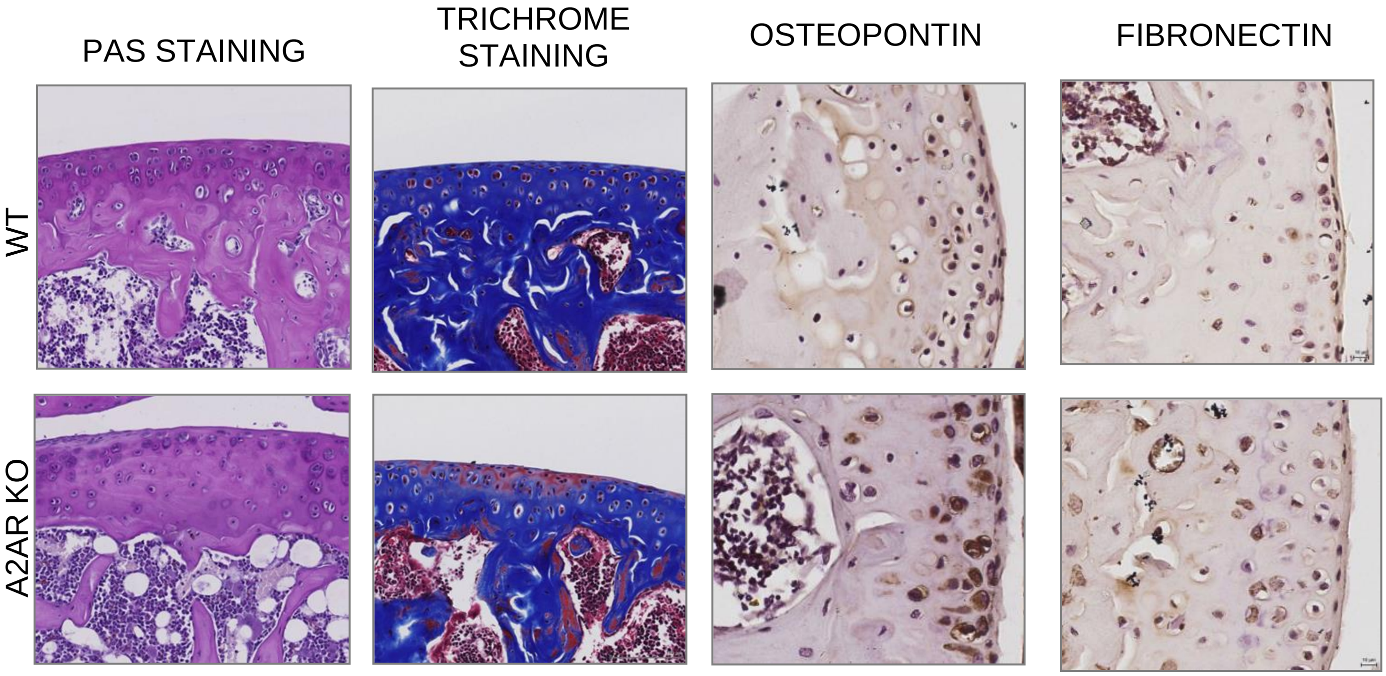

Supplementary Figure 2 - Representative photomicrographs of histological sections of the distal femoral condyles in WT and A2AR-KO mice at 12 weeks of age. Periodic acid–Schiff (PAS) staining and Trichrome staining show progressive reduction of glycogen (magenta staining) and collagen (blue staining) in the matrix of cartilage components in A2ARKO mice as compared to WT mice. On the right are representative photomicrographs of the distal femoral condyles in WT and A2AR-KO mice at 12 weeks analyzed for osteopontin and fibronectin by immunohistochemistry, as described.

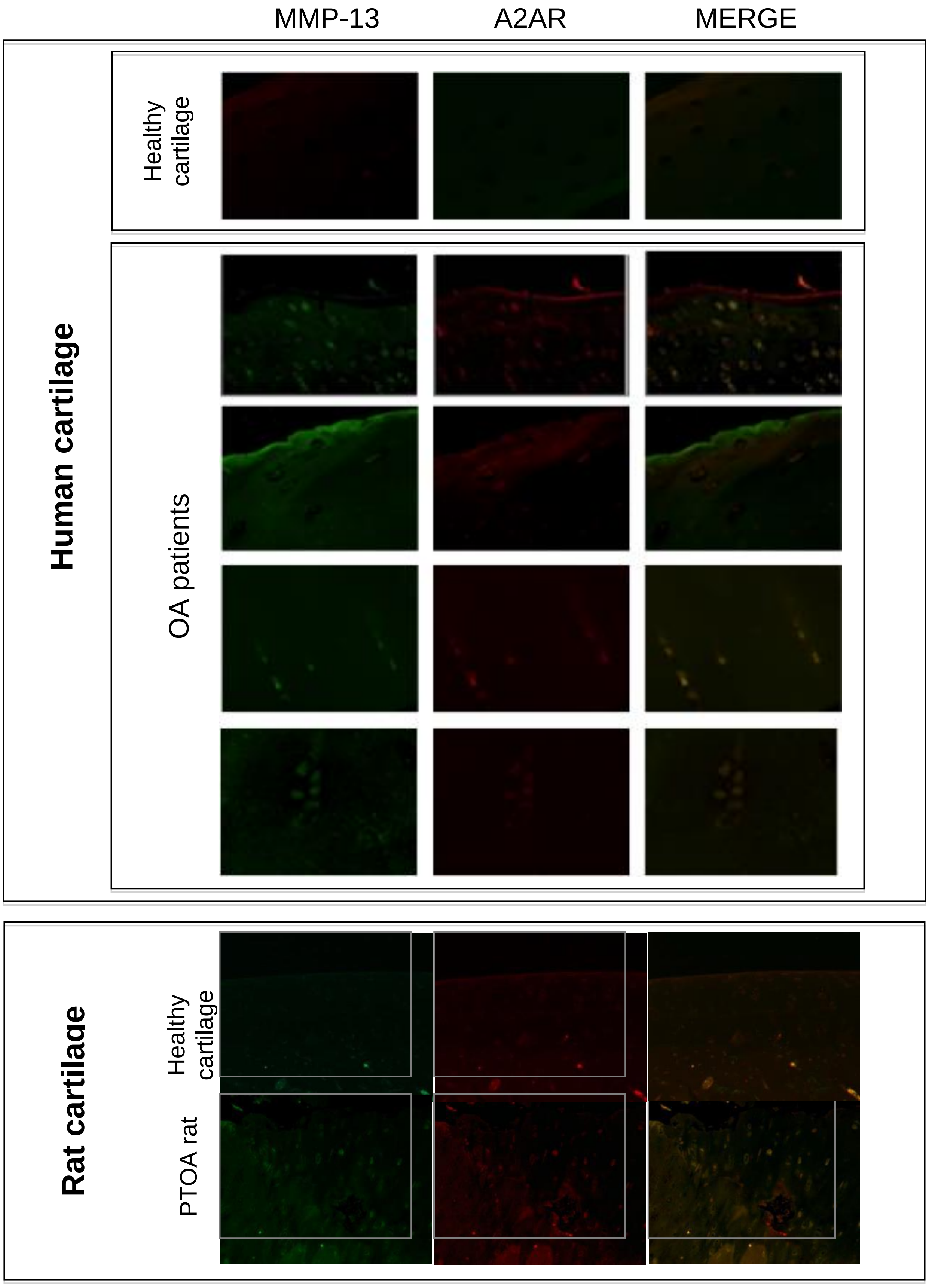
MMP-13
A2AR
MERGE
Human cartilage
Healthy cartilage
OA patients
Rat cartilage
Healthy cartilage
PTOA rat

Supplementary Figure 3 - A2AR is highly expressed in OA cartilage. Tissue sections from paraffin-embedded blocks of decalcified bone obtained from post-traumatic OA rats (bottom two rows) and from an OA patient at the time of joint replacement. MMP13 and A2AR are carried out as previously described. Shown are representative fields (original magnification 400X for human samples and 200X for rat samples).

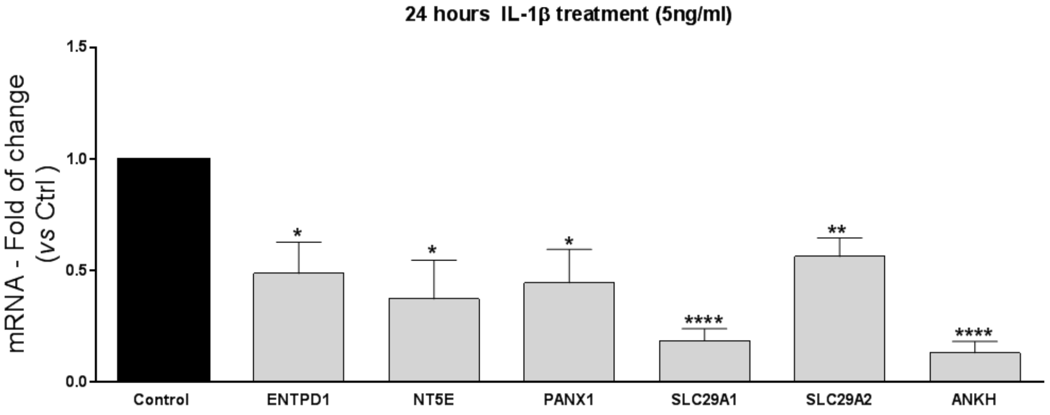

Supplementary Figure 4 - IL-1β treatment decreases expression of mRNA for enzymes and transporters involved in maintaining adenosine levels in the extracellular space. Mouse chondrocytes were isolated and treated for 24 hours with IL-1β (5ng/ml) before isolation of RNA, reverse transcription and quantitation by RT-PCR. There are significant decreases in ENTPD1, NT5E, PANX1, SLC29A1, SLC29A2 and ANKH mRNA. (n=4; data are represented as means ± SEM. *, $p<0.05$; **, $p<0.01$; ****, $p<0.001$ *vs* Control; Student's t test).

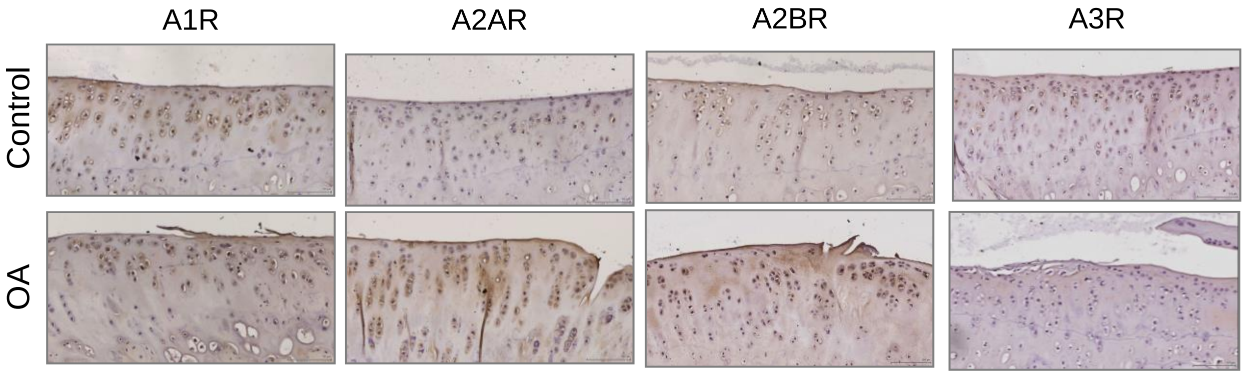

Supplementary Figure 5 - A2AR expression increases in chondrocytes of OA rats. Representative photomicrographs of adenosine receptor immunostaining of chondrocytes. A1R, A2AR, A2BR and A3R are expressed in rat articular cartilage. A2AR expression increases in chondrocytes of OA rats.

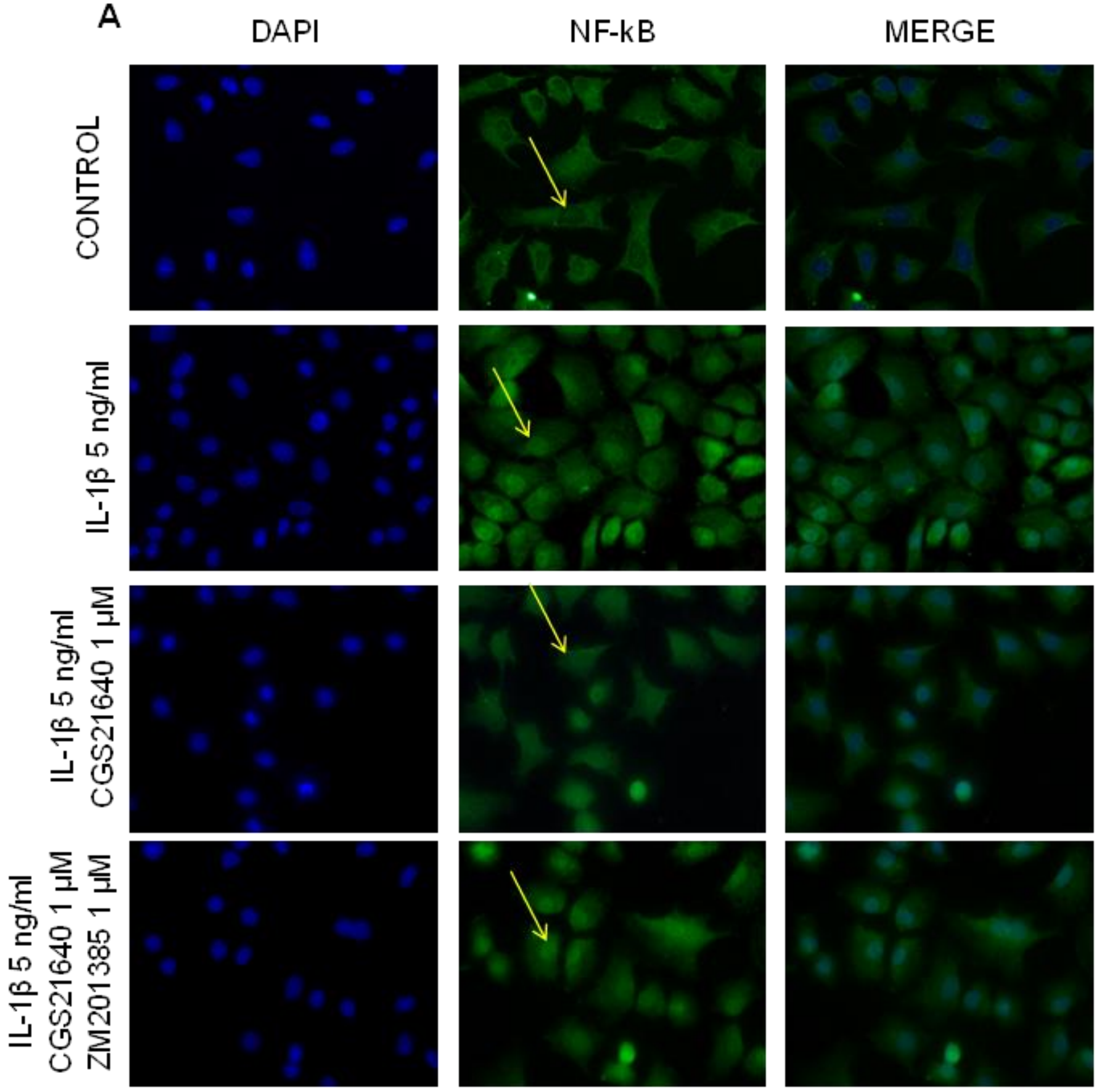
A
DAPI
NF-kB
MERGE
CONTROL
IL-1β 5 ng/ml
IL-1β 5 ng/ml
CGS21640 1 µM
IL-1β 5 ng/ml
CGS21640 1 µM
ZM201385 1 µM

B

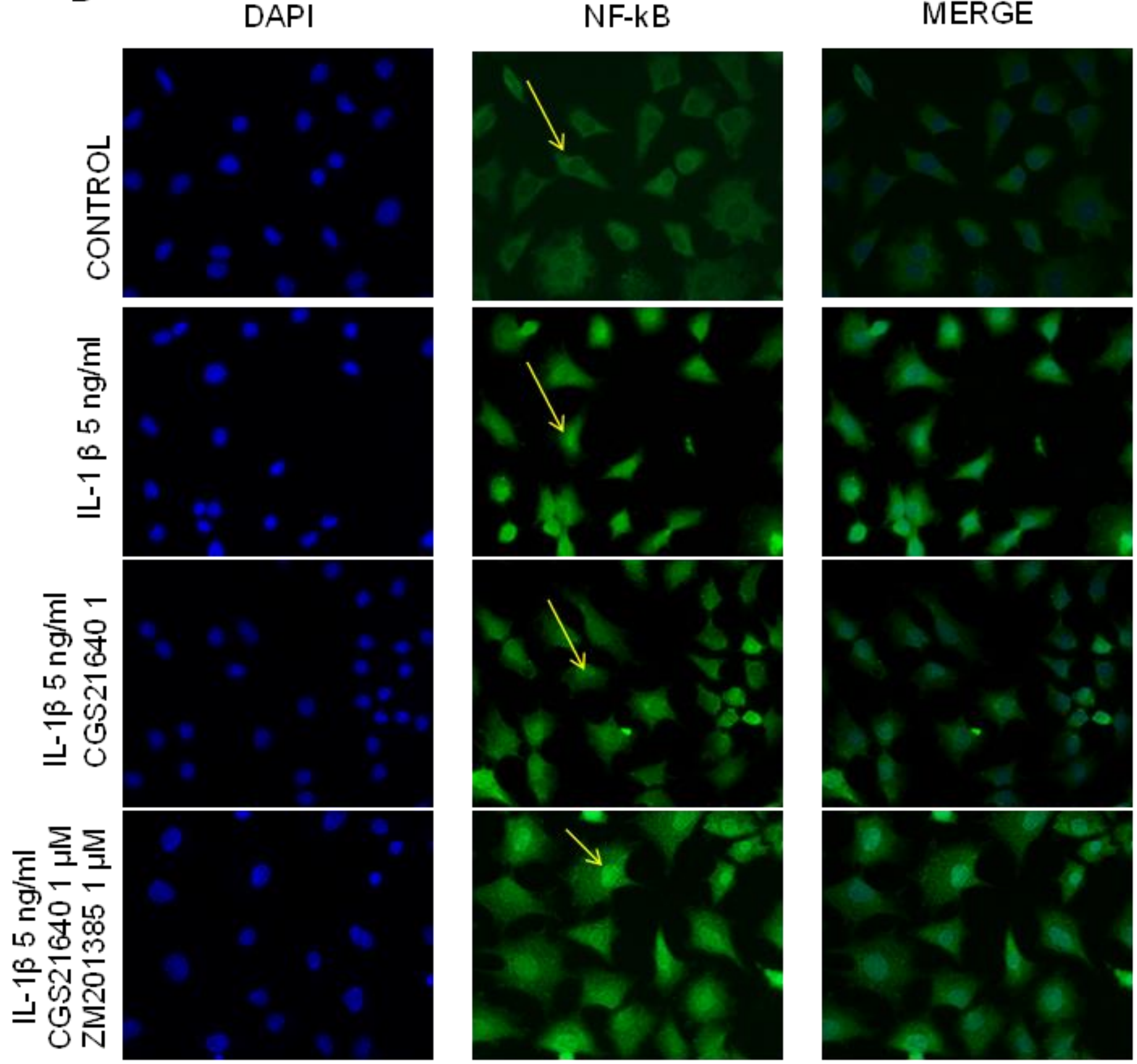

DAPI
NF-kB
MERGE
CONTROL
IL-1 β 5 ng/ml
IL-1β 5 ng/ml
CGS21640 1
IL-1β 5 ng/ml
CGS21640 1 μM
ZM201385 1 μM

C

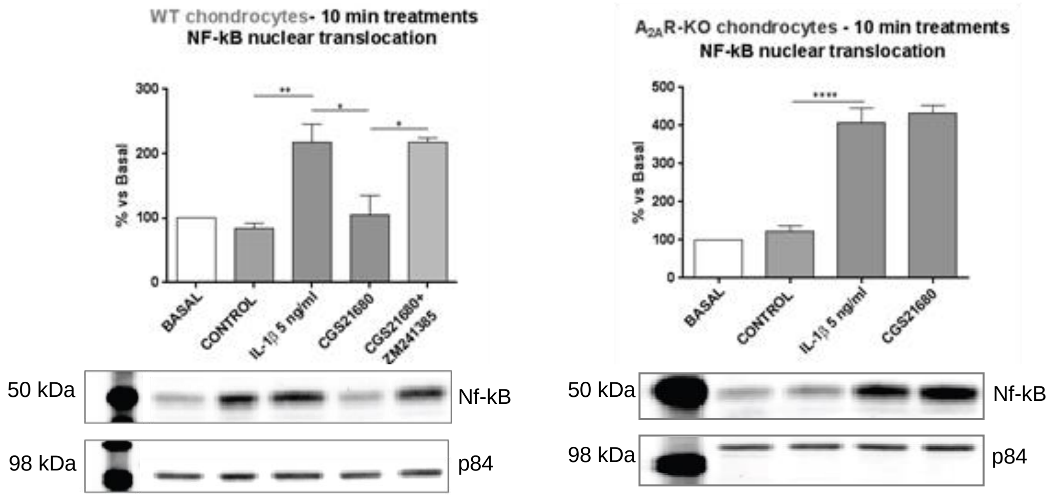

WT chondrocytes- 10 min treatments
NF-kB nuclear translocation
% vs Basal
0
100
200
300
BASAL
CONTROL
IL-1β 5 ng/ml
CGS21680
CGS21680+ ZM241385
50 kDa
Nf-kB
98 kDa
p84
A2AR-KO chondrocytes - 10 min treatments
NF-kB nuclear translocation
% vs Basal
0
100
200
300
400
500
BASAL
CONTROL
IL-1β 5 ng/ml
CGS21680
50 kDa
Nf-kB
98 kDa
p84

Supplementary Figure 6 - IL-1β induces NF-kB nuclear translocation in primary chondrocytes from WT and A2AR-KO mice. Chondrocytes were isolated from neonatal WT and A2AR-KO mice and cultured as described. Cells were fixed and were stained for NF-kB, as described, before examination under fluorescence microscopy. Shown are representative photomicrographs of (A) Chondrocytes from WT mice treated, as indicated. The yellow arrow points to the nucleus of cells. In (B) are shown photomicrographs of chondrocytes from A2AR-KO mice. (C) Western blotting and quantification of bands were performed on nuclear proteins from primary mouse chondrocytes after IL-1β (5ng/ml), CGS21680 (1μM), and ZM241385 (1μM) treatment (Data are represented as means ± SD; n=4 for each group; *, $P<0.05$; **, $P<0.001$ *vs* WT; one-way ANOVA followed by Bonferroni post-hoc test).

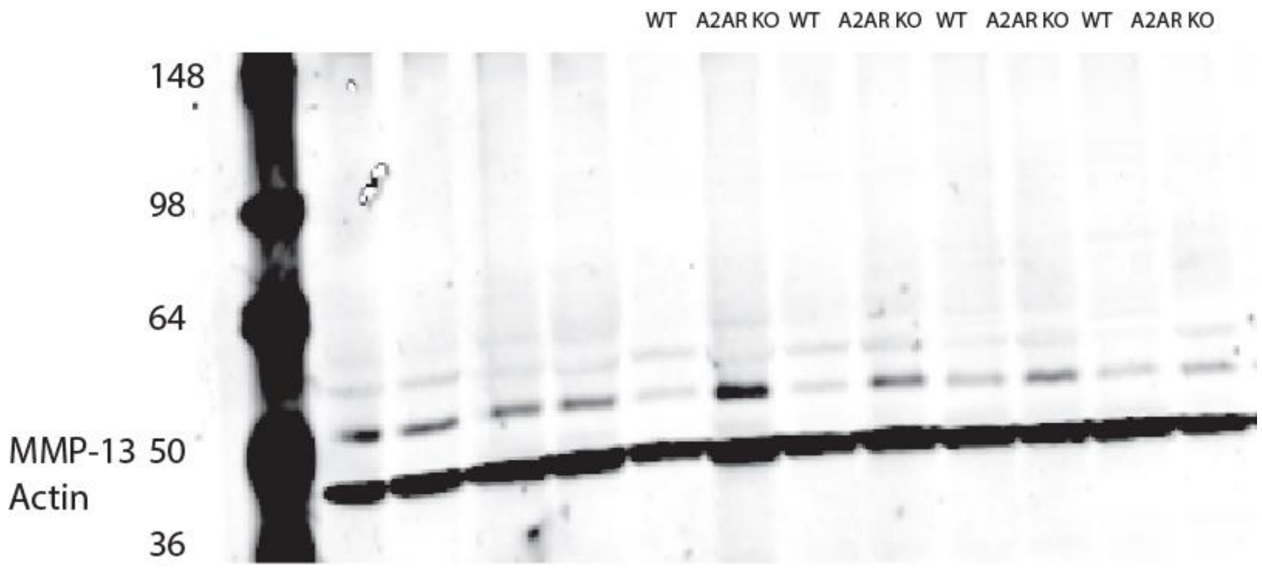

Supplementary Figure 7 - Western blot analysis for MMP-13 and actin. Experiment described in figure 2.

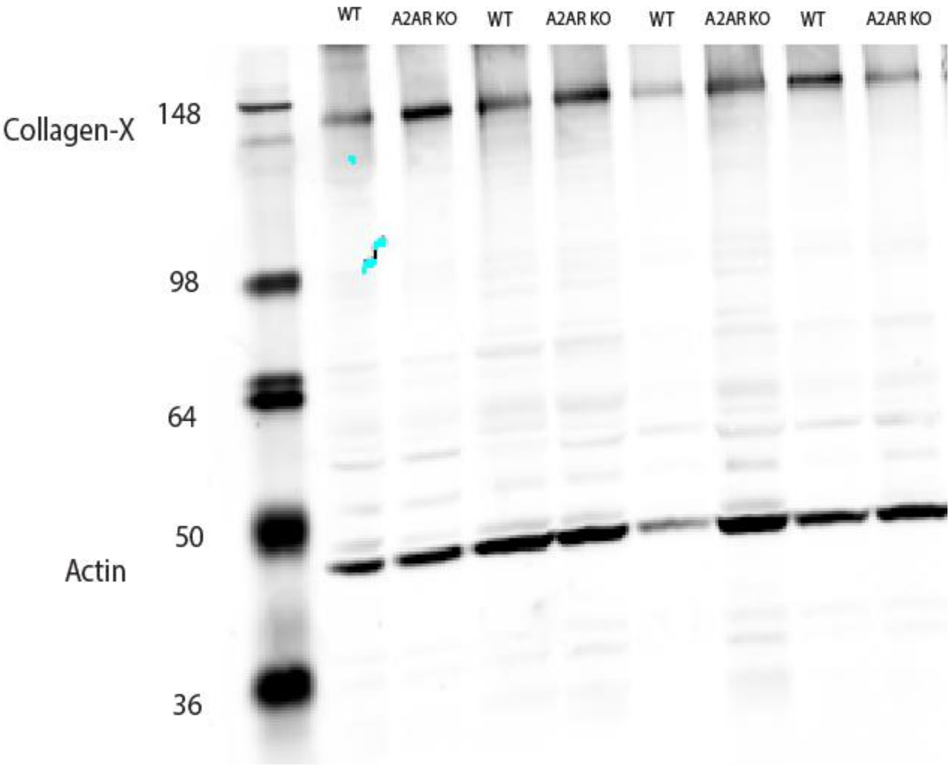

Supplementary Figure 8 - Western blot analysis for Collagen-X and actin. Experiment described in figure 2.

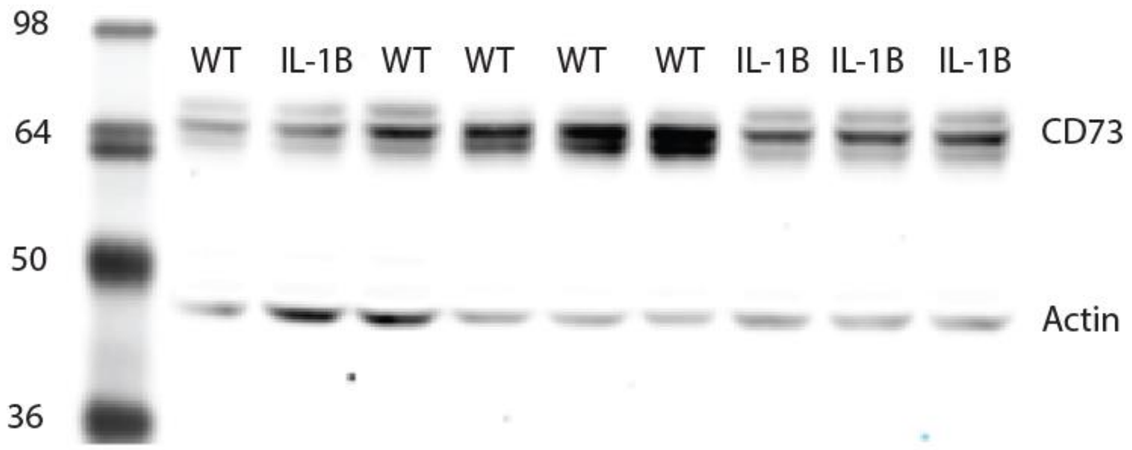

Supplementary Figure 9 - Western blot analysis for CD73-NT5E and actin. Experiment described in figure 4.

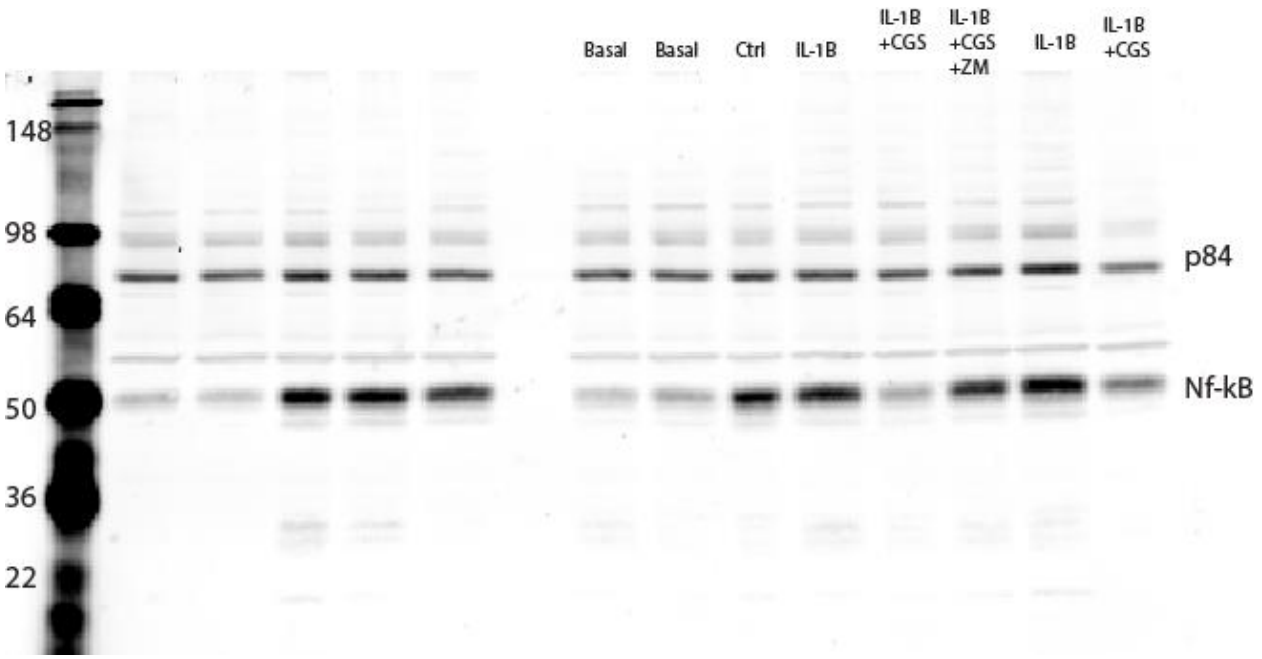

Supplementary Figure 10 - Western blot analysis for Nf-kB and p84. Experiment described in Supplemental figure 6 (WT chondrocytes).

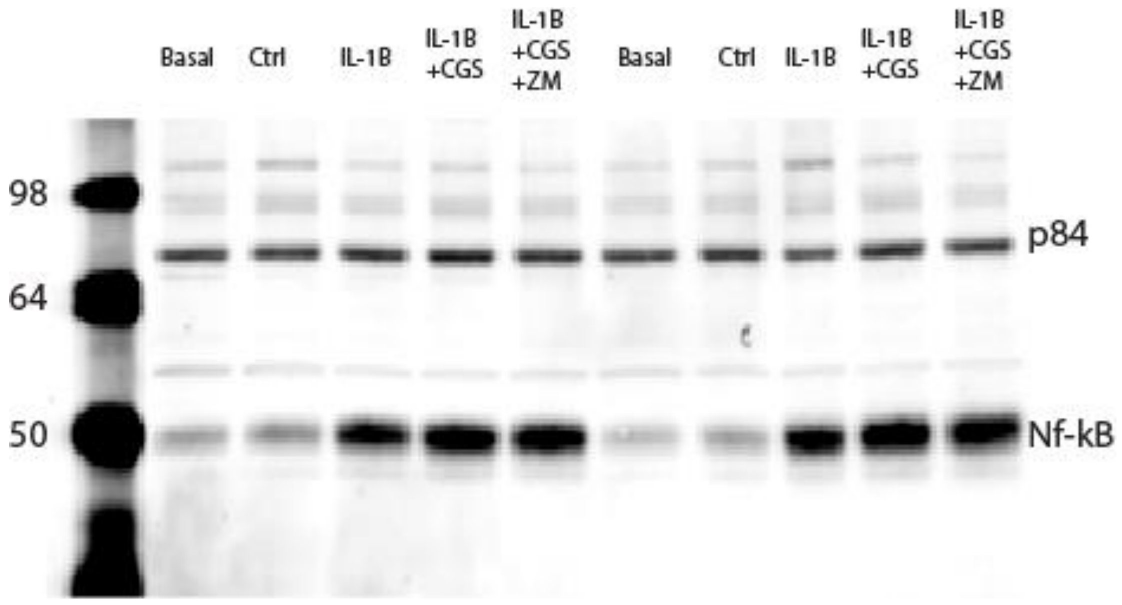

Supplementary Figure 11 - Western blot analysis for Nf-kB and p84. Experiment described in Supplemental figure 6 (A2ARKO chondrocytes).

		Age (weeks)	WT (mean±SD)	A2AR-KO (mean±SD)
BV	Femur	8	0.6785±0.0687	0.4907±0.0379**
		12	0.8912±0.1597	0.4524±0.0113***
		26	0.4678±0.0658	0.2784±0.0135**
		52	0.4375±0.1047	0.3041±0.1167
	Tibia	8	0.4032±0.0404	0.2998±0.0281**
		12	0.4848±0.0709	0.3651±0.0410**
		26	0.2836±0.0304	0.1838±0.0154**
		52	0.2013±0.0196	0.1640±0.0807*
BV/TV	Femur	8	12.530±0.720	10.480±0.534
		12	17.600±2.668	10.590±0.059***
		26	11.670±0.714	6.990±0.271***
		52	8.412±1.748	6.163±2.303
	Tibia	8	9.049±0.660	7.472±0.311
		12	13.600±1.761	10.7700±0.859**
		26	10.650±0.970	6.8060±0.359***
		52	6.383±0.689	5.175±2.447
Tr. Th.	Femur	8	0.0452±0.0014	0.0417±0.0003
		12	0.0611±0.0053	0.0421±0.0005***
		26	0.0478±0.0005	0.0454±0.0085
		52	0.0602±0.0014	0.0610±0.0062
	Tibia	8	0.0421±0.0008	0.0407±0.0008
		12	0.0547±0.0030	0.0483±0.0047**
		26	0.0478±0.0005	0.0454±0.0085
		52	0.0602±0.0014	0.0610±0.0062
Tr. N.	Femur	8	2.751±0.087	2.518±0.136*
		12	2.823±0.223	2.515±0.021**
		26	2.439±0.125	1.540±0.037**
		52	1.381±0.257	1.094±0.441
	Tibia	8	2.144±0.124	1.842±0.094*
		12	2.453±0.199	2.240±0.051
		26	2.294±0.190	1.506±.055***
		52	1.167±0.120	0.982±0.471
BMD	Femur	8	0.1594±0.0063	0.1405±0.0057
		12	0.2047±0.0237	0.1378±0.0016***
		26	0.1455±0.0088	0.0917±0.0053***
		52	0.1209±0.0169	0.0946±0.0243
	Tibia	8	0.1297±0.0056	0.1114±0.0033
		12	0.1711±0.0173	0.1414±0.0089**
		26	0.1354±0.0124	0.1052±0.0052**
		52	0.1033±0.0083	0.0897±0.0262

Supplementary Table 1 - μCT analysis of trabecular mid-shaft bone - Quantification of bone volume (BV), bone volume/tissue volume (BV/TV), trabecular thickness (Tb.Th.), trabecular number (Tb.N.) and bone mineral density (BMD) in tibia and femur of A2AR-KO and WT male mice at different ages (n=4 for each group. Data are expressed as mean±SD. *, $P<0.05$; **, $P<0.01$; ***, $P<0.001$ *vs* WT; Student's t test).

		Age (weeks)	WT (mean±SD)	A2AR-KO (mean±SD)
BMD	Femur	8	0.3475±0.0332	0.2966±0.0199*
		12	0.3924±0.0200	0.3258±0.0093**
		26	0.3656±0.0238	0.3593±0.0072
		52	0.3547±0.0195	0.3305±0.0223
	Tibia	8	0.3318±0.0314	0.3049±0.0270
		12	0.3979±0.0299	0.3632±0.0233*
		26	0.3951±0.0154	0.3754±0.0177
		52	0.3496±0.0115	0.3444±0.0213

Supplementary Table 2 - μCT analysis of subchondral bone - Bone mineral density in tibia and femur of A2AR-KO and WT male mice at different age (n=4 for each group. Data are expressed as mean±SD. *, $P<0.05$; **, $P<0.01$ vs WT; Student's t test).

		Age (weeks)	WT (mean±SD)	A2AR-KO (mean±SD)
BV	Femur	8	3.197±0.1049	2.673±0.1317**
		12	2.153±0.1899	1.605±0.1411**
		26	2.971±0.4243	2.506±0.110*
		52	1.955±0.505	1.974±0.2444
	Tibia	8	1.960±0.07712	1.6691±0.1161
		12	0.7101±0.1249	0.94±0.3138
		26	0.7201±0.09321	0.7689±0.09711
		52	0.7405±0.1365	0.7336±0.04032
BV/TV	Femur	8	56.370±2.796	50.400±2.089*
		12	59.100±4.055	51.210±1.463***
		26	55.960±2.289	55.470±1.087
		52	50.610±8.984	53.270±3.795
	Tibia	8	62.530±2.660	59.320±1.938
		12	61.430±6.895	54.060±1.781*
		26	53.640±1.366	54.550±3.017
		52	53.200±6.146	53.020±3.766
BMD	Femur	8	0.799±0.0169	0.791±0.005
		12	0.869±0.021	0.841±0.016*
		26	0.857±0.014	0.889±0.010*
		52	0.966±0.032	0.970±0.058
	Tibia	8	0.791±0.008	0.767±0.007*
		12	0.870±0.016	0.839±0.014**
		26	0.867±0.006	0.883±0.008*
		52	0.951±0.024	0.970±0.032

Supplementary Table 3 - µCT analysis of joint cortical bone- Quantification of bone volume (BV), bone volume/tissue volume (BV/TV), and bone mineral density in tibia and femur of A2AR-KO and WT male mice at different age (n=4 for each group. Data are expressed as mean±SD. *, P<0.05; **, P<0.01 vs WT; Student's t test).
